# Climate Change Experienced by Life on Earth—Drivers and Mechanisms of Future Trends in Microclimates

**DOI:** 10.1111/gcb.71140

**Published:** 2026-10-21

**Authors:** C. Greiser, E. Gril, I. M. D. Maclean, R. Beugnon, P. le Roux, J. Kemppinen, M. W. Rhodes, J. Aalto, D. M. Christiansen, P. De Frenne, S. Haesen, D. Klinges, M. Kopecký, J. Lenoir, M. Luoto, L. M. Mercado, M. Rantanen, G. Strandberg, L. van den Brink, J. Lembrechts, K. Van Meerbeek

**Affiliations:** ^1^ Department of Physical Geography and Bolin Centre for Climate Research Stockholm University Stockholm Sweden; ^2^ Department of Forest Ecology and Management Swedish University of Agricultural Sciences Umeå Sweden; ^3^ CNRS, Chrono‐Environnement (UMR 6249) Université Marie et Louis Pasteur Besançon France; ^4^ Environment and Sustainability Institute University of Exeter, Penryn Campus Penryn UK; ^5^ Centre for Ecology and Evolution University of Exeter, Penryn Campus Penryn UK; ^6^ German Center for Integrative Biodiversity Research (iDiv) Halle‐Jena‐Leipzig Leipzig Germany; ^7^ Institute of Biology Leipzig University Leipzig Germany; ^8^ CNRS, EPHE, IRD, CEFE Univ Montpellier Montpellier Cedex 5 France; ^9^ Ecology and Biodiversity, Department of Biology Utrecht University Utrecht the Netherlands; ^10^ Department of Plant and Soil Sciences University of Pretoria Pretoria South Africa; ^11^ Botany and Mycology Unit, Finnish Museum of Natural History University of Helsinki Helsinki Finland; ^12^ Department of Organismal and Evolutionary Biology, Faculty of Biological and Environmental Sciences University of Helsinki Helsinki Finland; ^13^ Climate Impacts and Adaptation Finnish Meteorological Institute Helsinki Finland; ^14^ Department of Geosciences and Geography University of Helsinki Helsinki Finland; ^15^ Department of Plant and Environmental Sciences University of Copenhagen Frederiksberg C Denmark; ^16^ Globe Institute University of Copenhagen Copenhagen K Denmark; ^17^ Forest & Nature Lab Gontrode Belgium; ^18^ Department of Earth and Environmental Sciences KU Leuven Leuven Belgium; ^19^ KU Leuven Plant Institute KU Leuven Leuven Belgium; ^20^ School of the Environment Yale University New Haven Connecticut USA; ^21^ Department of Ecology, Evolution, & Natural Resources Rutgers University New Brunswick New Jersey USA; ^22^ Institute of Botany of the Czech Academy of Sciences Průhonice Czech Republic; ^23^ Department of Forest Ecology, Faculty of Forestry and Wood Sciences Czech University of Life Sciences Prague Praha‐Suchdol Czech Republic; ^24^ UMR CNRS 7058 “Ecologie et Dynamique des Systèmes Anthropisés” (EDYSAN) Université de Picardie Jules Verne Amiens France; ^25^ Faculty of Environment, Science and Economy University of Exeter Exeter UK; ^26^ UK Centre for Ecology & Hydrology Wallingford UK; ^27^ Rossby Centre Swedish Meteorological and Hydrological Institute (SMHI) Norrköping Sweden; ^28^ Department of Thematic Studies—Environmental Change Linköping University Linköping Sweden; ^29^ Department of Ecosystem Management, Climate and Biodiversity, Institute of Botany BOKU University Vienna Austria; ^30^ Plants and Ecosystems (PLECO), Department of Biology University of Antwerp Wilrijk Belgium

**Keywords:** biosphere‐atmosphere interactions, buffering, clouds, cold‐air pooling, decoupling, evapotranspiration, surface‐energy balance, vegetation‐climate feedbacks

## Abstract

Microclimate, the climate directly experienced by organisms, is crucial for understanding ecological responses to climate change, yet its long‐term dynamics and the drivers of decadal microclimate changes remain underexplored in ecology and global change biology. Microclimate changes do not necessarily track macroclimate trends. Changes in meteorological conditions (e.g., cloud cover, wind patterns) and vegetation dynamics (e.g., canopy opening, desertification in drylands, shrub encroachment in the tundra) associated with climate change can lead to locally divergent microclimate trends, thereby altering spatial variability. Understanding and quantifying how macroclimate change translates to fine‐scale microclimate change experienced by organisms, populations and communities is therefore essential for predicting ecological responses to global environmental change. This review examines how macroclimate change drives microclimate changes as experienced by life on Earth—and how these micro‐scale changes may, in turn, feed back into the regional and global climate system. Our primary focus is on near‐surface air and soil temperature—due to their relevance to most terrestrial organisms and ecosystem processes. The review is structured around three pathways of microclimate changes: (I) direct effects of changes in meteorological conditions, (II) indirect effects via vegetation changes, and (III) feedbacks between microclimate and climate change. Using three mechanistic model simulations in real‐world landscapes, we illustrate and quantify the key mechanisms that lead to divergent warming trends. We show that microclimate change emerges from interacting atmospheric, land‐surface, and biophysical processes, which can differ substantially across space and time. Predicting future microclimates therefore requires accounting for these processes and their temporal dynamics. By providing an accessible synthesis of these processes, this review aims to strengthen links between ecology and atmospheric science and improve our understanding of how climate change translates into microclimatic conditions relevant to life on Earth.

## Graphical abstract

Microclimate changes do not necessarily track macroclimate trends. However, changes in meteorological conditions (e.g., cloud cover) and vegetation dynamics (e.g., shrub encroachment) associated with climate change can lead to locally divergent microclimate trends, thereby altering spatial variability. Understanding and quantifying how macroclimate change translates to fine‐scale microclimate change experienced by organisms, populations and communities is essential for predicting ecological responses to global environmental change. This review examines how macroclimate change drives microclimate changes as experienced by life on Earth—and how these micro‐scale changes may, in turn, feed back into the regional and global climate system.

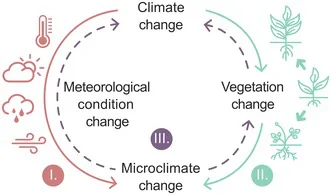

## 1 Introduction

Climate change impacts species worldwide (Pörtner et al. 2021). Shifts in temperatures, precipitation, and the frequency and severity of extreme weather events are already affecting the phenology, behavior, and distribution of organisms as well as the functioning of entire ecosystems (Grimm et al. 2013; Muthukrishnan et al. 2025; Soifer et al. 2025), with even greater impacts projected for the coming decades (IPBES 2019). However, climate change projections are primarily based on global and regional climate models that operate at coarse spatial resolutions (typically 10–100 km), which fail to capture the microclimatic conditions experienced by organisms (Christiansen et al. 2024; Haesen, Lenoir, et al. 2023; Klinges et al. 2024; see definitions in Box 1). As a result, failing to account for the microclimatic conditions experienced by organisms remains an important limitation when predicting biological responses to climate change.

Microclimates are shaped by interactions between atmospheric conditions and the Earth's surface, with local properties such as topography, vegetation, soils and water bodies modifying the exchange of energy and moisture (see Boxes 1 and 2), creating fine‐scale heterogeneous environments that can differ substantially—both spatially and temporally—from macroclimate averages, which represent broader geographic regions (Geiger 1966). In this review, we focus on microclimate change—defined as long‐term (decadal or longer) changes in near‐surface temperature. Importantly, microclimate changes do not always mirror macroclimate changes. Shifts in meteorological conditions directly influence microclimate warming rates, or indirectly do so by altering vegetation structure and composition (Seidl et al. 2017). In turn, changes in land surface properties can feed back on meteorological conditions through multiple biophysical processes, including vegetation‐climate interactions (Duveiller et al. 2021), further influencing the extent to which microclimate change tracks macroclimatic trends (Luo et al. 2024a; Miralles et al. 2025). The decoupling between microclimate and macroclimate changes leads to spatially and temporally variable differences between microclimate and macroclimate. Using a mechanistic modeling approach, Maclean (2020) reconstructed daily maximum near‐ground temperatures over approximately three decades, revealing divergent temperature trends among locations within a 2 × 2 km landscape in the UK (Figure 1). Beyond the microclimate scale, spatial heterogeneity in warming rates also emerges at the mesoscale, for instance through variations in elevational lapse rates, coastal–inland gradients, and continentality effects. These mesoclimatic processes set the climatic context for microclimates and can lead to stronger or weaker local warming, thereby influencing the magnitude and direction of micro‐macroclimate decoupling.

**FIGURE 1:**
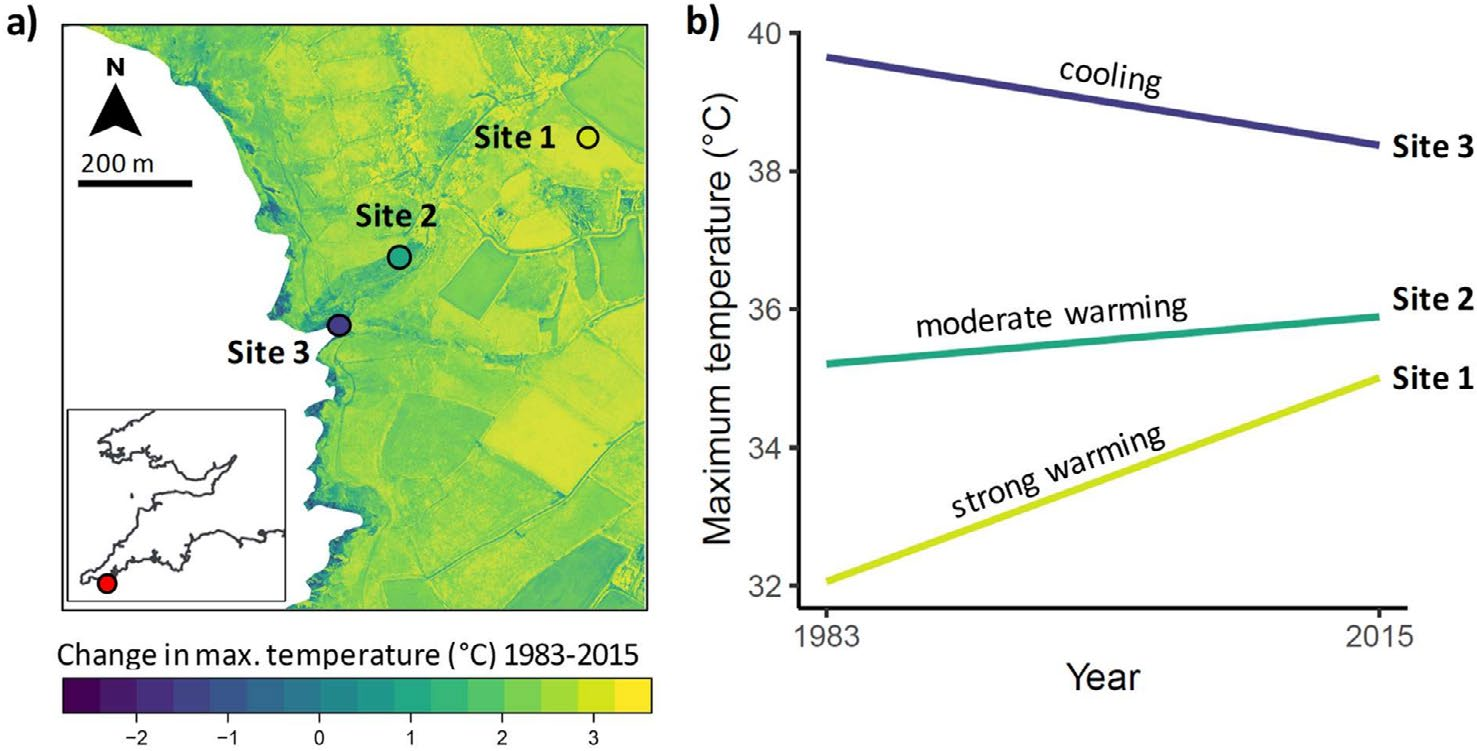
Change in microclimate maximum temperatures at Caerthillian Cove, Lizard Peninsula, UK (49.97° N, 5.21° W), from 1983 to 2015. Data were generated using a mechanistic microclimate modeling approach (Maclean 2020). Microclimate temperature changes were derived for each grid cell by linear regression on annual maximum air temperature (5 cm above ground). Interactions between terrain, vegetation, and weather caused local warming rates to diverge from the regional macroclimate trend (0.3°C over the ~3 decades). Three example locations illustrate strong warming (2.9°C), moderate warming (0.7°C), or even cooling (−1.3°C).

Despite their importance, microclimates are still often overlooked in climate change impact assessments. Yet it is these proximal conditions that determine how individual organisms, populations, and ecosystems respond to environmental change (Kemppinen et al. 2024; Kemppinen, Niittynen, le Roux, et al. 2021; Körner and Hiltbrunner 2018). Ignoring the fine‐scale variability in microclimate trends risks misjudging both the vulnerability and resilience of species and ecosystems to climate change (Niittynen et al. 2018). By integrating microclimate change into ecological research, we can improve predictions of species distributions (Haesen, Lenoir, et al. 2023; Maclean and Early 2023), physiological responses (Buckley et al. 2023; Kearney and Leigh 2024), and ecosystem functioning (Beugnon et al. 2024; Virkkala et al. 2024) under climate change.

Here, we review how and why microclimate change differs from macroclimate change, and explore the drivers and feedbacks underlying these dynamics. We focus on three key pathways of microclimate change (Figure 2): (I) changing meteorological conditions; (II) physiological, structural and compositional vegetation responses to climate change; and (III) feedbacks between microclimate and climate change. While our primary focus is on microclimate temperature, we also discuss other key microclimate components—soil moisture, air humidity and radiation—as these influence many ecological processes and also interact with temperature. We deliberately exclude direct anthropogenic land‐use and land‐cover change to isolate the effects of climate change on microclimate, while acknowledging that these human‐driven changes remain important. To better capture biologically relevant thermal conditions, we emphasize daily and seasonal extremes instead of mean temperatures, which can obscure ecologically important variability and asymmetric changes between day and night or between seasons (Helmuth et al. 2010; Cox et al. 2020; Shi and Wu 2025).

**FIGURE 2:**
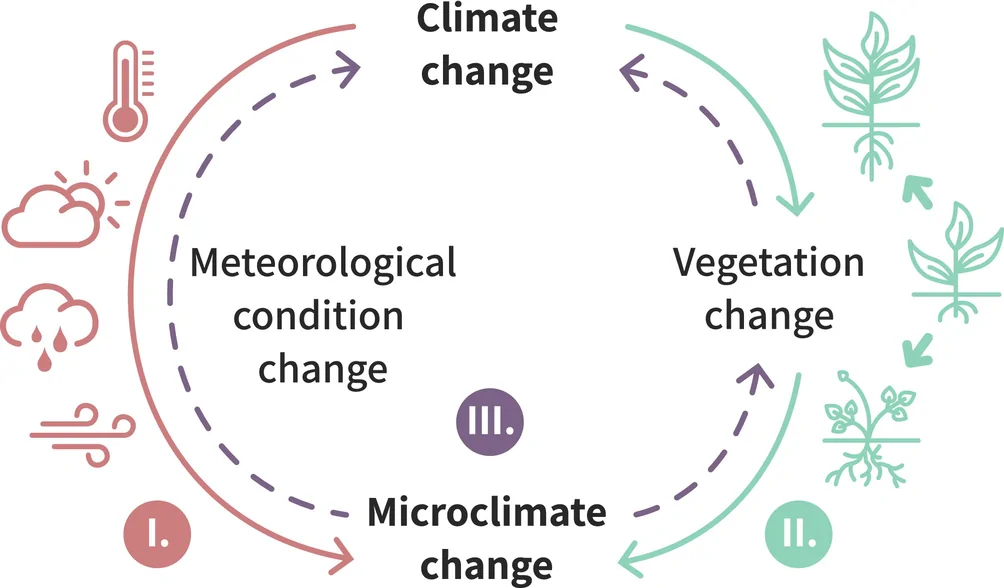
Conceptual figure visualizing how this review is structured around three pathways of microclimate change: (I) direct effects of changes in meteorological conditions (red), (II) indirect effects via vegetation changes (green) and (III) feedbacks between microclimate and global climate change (purple, dashed arrows).

To illustrate the mechanisms that drive variation in microclimate change, we ran three different case studies of model simulations using well‐validated mechanistic microclimate models (*microclimf* and *micropoint*; Maclean 2026) with air temperatures 5 cm above ground as the response variable. Further details to all case studies are provided in Boxes 3 and 4 and Supporting Information A.

Rather than providing a comprehensive treatment of atmospheric physics, this review is intended as a bridge for ecologists to the physical principles needed to understand, measure, and model microclimate change. In addition, it is intended as a bridge for atmospheric scientists and land‐surface modellers to the ecological processes underlying microclimate and macroclimate change. By translating key insights from atmospheric science into an ecological context, and by highlighting that microclimate is strongly shaped by the complexity of the biosphere, we hope to foster stronger cross‐disciplinary understanding and ultimately improve projections of biodiversity change and ecosystem management under rapid climate change.

BOX 1 **Glossary.**

**Adiabatic lapse rate**: The decrease in temperature of a parcel of air that rises in the atmosphere without exchanging heat or mass with the surroundings. Rising air expands as atmospheric pressure decreases and therefore cools, whereas descending air is compressed and warms.

**Leaf Area Index (LAI)**: The total one‐sided leaf area per unit ground area for broadleaf species, or projected hemi‐surface leaf area for needleleaf species.

**Macroclimate**: Climatic conditions as measured by synoptic weather stations in locations chosen to minimise the influence of local surface features such as vegetation, soil properties, and fine‐scale topography. The term refers to the general climatic conditions experienced across the broader landscape, rather than local microclimatic variation. Spatial scale: kilometres to continental/global scale.

**Mesoclimate**: The climate of an intermediate spatial scale, typically covering areas such as valleys, hillsides, or entire landscapes, situated between microclimate and macroclimate. It is impacted by meso‐scale topographic features, land cover, distance to coastlines and local weather systems (Barry and Chorley 2009). Spatial scale: ~1 km to several tens or a few hundred km.

**Microclimate**: The climate in a small area near the Earth's surface that is shaped by local surface features such as vegetation, soil properties, water bodies, and fine‐scale topography. The term refers to local climatic conditions that may differ substantially from the surrounding meso‐ or macroclimate and that often directly influence organisms and ecosystem processes (Barry and Blanken 2016; De Frenne et al. 2025). See also *proximal microclimate*. Spatial scale: ~1 cm to hundreds of metres.

**Microclimate buffering**: The decrease in amplitude of diurnal or seasonal temperature fluctuations compared to reference macroclimate fluctuations. While microclimate buffering leads to a lower amplitude, for example under tree canopies, microclimate amplification leads to a larger amplitude.

**Proximal microclimate**: The microclimate close (proximal) to an organism, population or ecosystem of interest (Klinges et al. 2024). While earlier definitions of microclimate refer to near‐ground climate at high temporal and spatial resolution, the term proximal climate emphasizes the importance of biological/ecological relevance (proximity) of the climate to an organism in models linking climate and ecological processes/patterns. For instance, the proximal microclimate for an arboreal frog would be the climatic conditions in the tree canopy, whereas the proximal microclimate for an earthworm would be soil temperature and moisture conditions in the upper 10 cm of the soil.

**Vapor Pressure Deficit (VPD)**: VPD is the difference between the *saturation vapor pressure* (i.e., water vapor pressure in fully saturated air at a given temperature) and the *actual water vapor pressure* in the air. In other words, it quantifies the potential for evaporative water loss (Jones 2014).

BOX 2 **The physics of microclimate.**

Microclimates are created through exchanges of energy between the ground, water, vegetation, and the air (Figure 3). Incoming solar (shortwave) radiation, that is, sunlight, and thermal (longwave) radiation are partitioned into energy that warms surfaces and the air (sensible heat), drives evaporation and other phase changes (latent heat), or is stored in the ground and vegetation. These energy exchanges determine surface and near‐surface air temperatures (Chen et al. 1999; Oke 1987).

**FIGURE 3:**
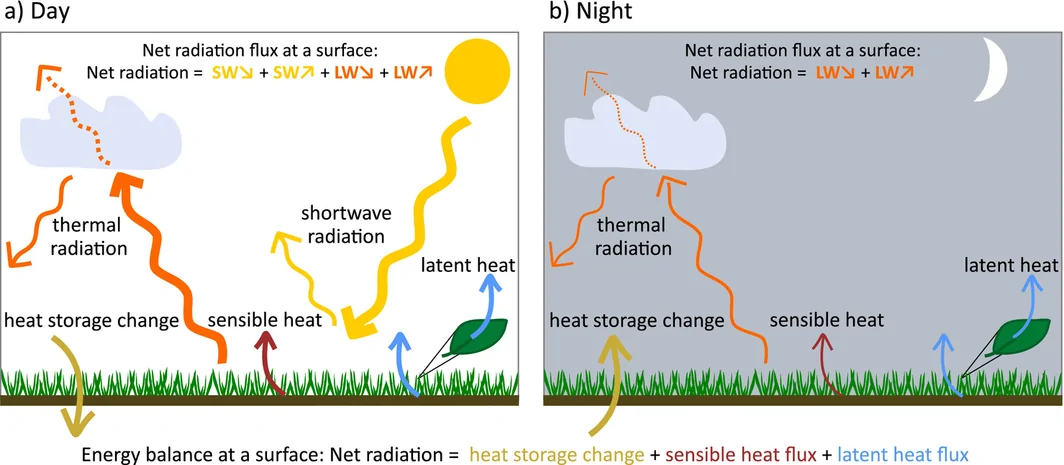
Main components of the energy exchange between the Earth's surface, vegetation, and atmosphere that shape microclimate variation across space and time (here for simplicity ignoring lateral heat transfer, such as cold‐air pooling). Microclimate conditions arise from the balance between incoming shortwave (SW) radiation and outgoing longwave (LW) radiation, and from the partitioning of the net radiation into sensible heat, latent heat, and heat storage change. Net radiation is usually positive during the day (a) and negative during the night (b). The radiation balance and energy fluxes are influenced by meteorological conditions—such as clouds or air humidity—and by land surface features—such as topography, vegetation structure, and soil moisture—thereby mediating local microclimatic variability and its divergence from broader macroclimate conditions.

Net radiation is the sum of incoming and outgoing radiation, comprising both shortwave radiation and longwave radiation. Radiation is either absorbed or reflected by surfaces or partially transmitted through vegetation, with dense canopy reducing the amount of solar radiation that reaches the ground. Natural surfaces mainly absorb incoming shortwave radiation from the sun and re‐emit energy as longwave radiation. Net radiation is typically positive during the day and negative at night but generally exhibits a smaller diurnal range beneath canopies than above them, contributing to microclimate buffering (Baldocchi and Vogel 1996; Ross 1975). Net radiation is further partitioned into sensible heat flux, latent heat flux, and heat storage change (Figure 3). Sensible heat, which results in a change of temperature of a substance, represents the energy transferred from warm surfaces to the overlying air by conduction and convection. Conduction transfers heat across the immediate surface boundary, whereas convection transports heat through air movement driven by buoyancy or wind. Latent heat is the energy absorbed or released during a phase change of water, such as evaporation, transpiration, condensation, freezing or melting, without changing temperature.

The rate of sensible and latent heat exchange depends on temperature or vapor pressure gradients and on wind speed, which enhances turbulent transport and thereby increases the exchange of heat and moisture between the surface and the atmosphere (Lewis and Randall 1923). These exchanges occur within the atmospheric boundary layer, the lowest part of the atmosphere that is directly influenced by the Earth's surface through exchanges of heat, moisture, and momentum (Oke 1987; Stull 1988). Latent heat transfer is additionally regulated by stomatal opening in response to radiation, vapor pressure deficit (VPD), temperature, CO_2_ concentration, and plant water status. Wind speeds are generally lower near the ground because of surface drag and are strongly influenced by terrain and vegetation. Thus, in forest understories and other sheltered environments, sensible and latent fluxes are reduced due to lower wind speeds and suppressed turbulence, contributing to the thermal decoupling of below‐canopy microclimates from above‐canopy macroclimatic conditions (Baldocchi and Vogel 1996; Finnigan 2000).

These fluxes are temperature‐dependent, and surface temperature can thus be estimated by assuming an equilibrium state is reached. In reality, the amount of heat stored in the ground increases during the day and decreases at night. Similarly, soils generally gain heat in spring and summer and lose heat in autumn and winter, causing subsurface temperatures to lag behind changes in air temperature. Energy storage in soil, vegetation and water forms an important component of the surface energy balance because not all absorbed energy is immediately transferred as sensible or latent heat, delaying warming and cooling over daily and seasonal timescales (Choudhury et al. 1987). Soil water content has a pronounced effect on rates of soil heat storage as wetter soils have higher thermal conductivity and specific heat capacity, and thus wet soils will typically store more heat, potentially damping temperature fluctuations near the soil surface (Campbell and Norman 2012).

Beyond localised energy fluxes, mesoclimate‐scale processes shape fine‐scale climatic variation through the lateral movement and mixing of air across landscapes (Barry and Chorley 2009). Elevation affects temperature via the **adiabatic lapse rate**, whereby air cools with altitude as pressure decreases (Figure 4a; Barry 2008; Lookingbill and Urban 2003). On clear, calm nights, dense air can accumulate in topographic depressions, producing cold‐air pooling, temperature inversions and frost pockets (Figure 4b). Proximity to water bodies also moderates temperature extremes, as water's high heat capacity slows heating and cooling, with inland influence depending on wind, topography, and surface roughness. Thus, both meso‐ and micro‐scale processes generate complex spatial patterns in near‐surface climate and warming rates.

**FIGURE 4:**
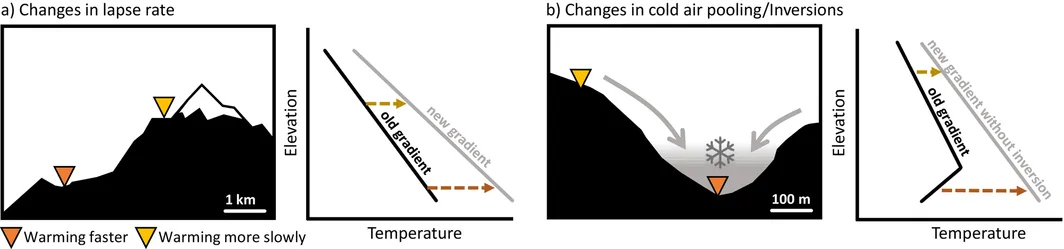
Conceptual figure of different rates of warming caused by changes in lapse rate and cold‐air pooling/inversion. (a) The black line shows a normal lapse rate, which is typical for the mixed troposphere. The right grey line represents a steeper lapse rate (i.e., temperatures decline faster with height), which can occur under conditions of enhanced surface warming (e.g., due to drying). As the lapse rate steepens, more of the warming is concentrated near the surface, leading to stronger warming at low elevations. This illustrates how identical atmospheric warming can produce different surface warming rates. The opposite pattern has also been observed, where mountain tops are warming faster than valleys (Pepin et al. 2025). (b) The black line shows a typical temperature profile during an inversion. If the conditions under which inversions form become less frequent (grey line), sites in different elevations may experience different rates of warming.

## 2 PART I: Changing Meteorological Conditions

In this section, we discuss how changes in temperature, lapse rates, near‐surface air temperature inversions, cloud cover, precipitation, humidity, snow cover, and wind alter microclimate landscapes and cause nearby microclimates to diverge (increasing spatial heterogeneity) or converge (microclimatic homogenization) through time. In order to illustrate single mechanisms, we discuss meteorological variables one by one and present their simulated effects on microclimate independently from other variables (Box 3). In reality, however, many of these variables change in parallel (e.g., cloud cover, air humidity, rainfall and soil moisture) and realistic rates of microclimate change can only be predicted by modeling the changes in all variables in concert (Figure 1; Maclean 2020).

### 2.1 Macroclimate Temperature

#### 2.1.1 General Increase of Air Temperature

As macroclimate air temperatures rise, surface temperatures usually also increase. Warmer surfaces emit more longwave radiation, which acts as a negative radiative feedback by partly counteracting further surface warming. The extent to which warming is buffered or amplified in near‐ground microclimates depends on how available energy is partitioned among sensible, latent, and ground heat fluxes. Rising temperatures also increase vapor pressure deficit (VPD) and consequently potential evapotranspiration (Clapeyron 1834; Clausius 1850), although actual evapotranspiration depends on soil moisture and vegetation response (Seneviratne et al. 2010). In moist sites, evaporation and transpiration can increase with increasing temperature and VPD, and latent heat flux further slows down surface warming. However, enhanced evaporation and transpiration also accelerate soil drying. As VPD rises and stomata close to limit water loss, evapotranspiration declines and a larger share of energy is converted to sensible heat, leading to accelerated surface warming. These mechanisms thus create potentially divergent warming trajectories between wet and dry microenvironments (Seneviratne et al. 2010; Srivastava et al. 2024). For example, García‐García et al. (2023) found that soil temperatures during annual heat extremes were rising faster than air temperatures (on average by 0.7 °C/decade) in two‐thirds of their European measurement sites. They attributed this faster rate to declining soil moisture and associated heat‐amplifying feedbacks, such as reduced latent heat transfer and the lower specific heat capacity of dry soil. Additionally, land generally warms 60% faster than oceans (Wallace and Joshi 2018) and is predicted to continue to do so (Byrne and O'Gorman 2018), causing coastal regions to warm more slowly than inland areas (Al‐Ruzouq et al. 2022; IPCC 2023a). In both cases, the difference between wet and dry sites or the difference between land and water can potentially lead to different rates of warming, increasing micro‐ and mesoclimate variability across landscapes.

#### 2.1.2 Lapse Rates and Their Role in Mesoclimate Warming Patterns

The adiabatic lapse rate—the rate at which air temperature decreases with altitude—plays a critical role in determining how warming is distributed vertically through the atmosphere and how much of that warming is expressed at the surface. In the troposphere, the lower layer of the atmosphere, temperature typically declines with altitude as rising air expands and cools under lower pressure. The actual rate of decline, however, depends on the water vapor content of the air: in completely dry air, the adiabatic lapse rate approaches 9.8°C/km, while in moist air condensation releases latent heat and reduces the rate of cooling with altitude. Because water vapor content varies across regions and over time, lapse rates are not uniform (Minder et al. 2010; Rolland 2003), and this means that neighboring sites, for example, those at different elevations, can experience different rates of microclimate warming even under the same regional macroclimate (Figure 4a). For instance, Chan et al. (2024) used the laws of thermodynamics to map the adiabatic lapse rate across all mountain regions globally. By combining this global map with data on temporal rates of change in mean annual temperature during the period 1971–2020, they mapped the vertical velocities of isotherm shifts across mountain regions. Reported values of vertical climate velocities ranged from 1.8 to 10.8 m/year. These spatial, and potentially temporal, variations in the lapse rate can either speed up or slow down surface warming, depending on the region, season, and local climatic conditions (Figure 4a). In the Mediterranean, stronger near‐surface summer warming has partly been attributed to steeper lapse rates associated with drier conditions. Modeling experiments show that not accounting for lapse‐rate changes reduces projected end‐of‐century Mediterranean summer warming by about 1°C (Brogli et al. 2021).

Changes in the lapse rate contribute to elevation‐dependent climate change. Globally, mountain tops warm on average 0.21°C per century faster than nearby lowlands, even if free‐air warming is spatially uniform (Pepin et al. 2025). However, regional exceptions occur. Additionally, in areas with complex topography, vertical mixing of air masses becomes more important, and altered lapse rates can influence the stability of the lower atmosphere, affecting both temperature inversions and the likelihood of cold‐air pooling (see below and Figure 4b). Therefore, changes in the lapse rate are a key atmospheric mechanism contributing to spatial heterogeneity in warming rates at mesoclimate scales.

#### 2.1.3 Temperature Inversions and Cold‐Air Pooling

Temperature inversions—where air temperature increases with altitude—are an important mechanism influencing near‐ground temperatures. Surface inversions typically form during calm, clear‐sky nights when strong radiative cooling of the ground cools the adjacent air, resulting in a layer of dense cold air beneath warmer air aloft. Although inversions may also arise from advective or synoptic processes, nocturnal radiative cooling dominates near‐surface inversions relevant to microclimates. The strength (temperature gradient) and depth (vertical extent) of these surface inversions determine how much they insulate the ground from warmer overlying air. Climate change is altering the frequency and characteristics of these inversions. A global trend toward shallower inversions, with regional level variable changes in inversion strength has been observed between 1989 and 2019 (Zeng et al. 2022). In the Arctic, a particularly strong decline in inversion depth has been observed and projected in winter months—due to the combined effects of sea ice loss and increased cloud cover, both of which reduce surface radiative cooling (Ruman et al. 2022; Zhang, Furtado, et al. 2021). According to Ruman et al. (2022), the largest wintertime changes in inversion strength are projected to occur over the Arctic Ocean, with increases of roughly 3°C–4°C under the RCP4.5 scenario by 2070–2099. Over Arctic land areas, the projected changes are notably smaller.

More localized near‐surface air temperature inversions are caused by cold‐air drainage and pooling, a process in which cooler, denser air flows downslope under gravity and accumulates in surface depressions (Figure 4b). This pooling can lead to strong and frequent near‐surface inversions, particularly in mountainous or forested areas (Daly et al. 2010). Cold‐air pooling is therefore best understood as a specific, landscape‐driven subset of surface inversions, one that is tightly coupled to local topography and land cover. For instance, under forest canopies, downslope cold‐air drainage can persist during the daytime thanks to the decoupling between canopy and sub‐canopy microclimatic conditions (Pypker et al. 2007), while in treeless areas, cold‐air drainage occurs typically only during the nighttime. Rupp et al. (2020) demonstrated that vertical temperature gradients within a mountain valley ranged between −10 °C/km and over 60 °C/km, with substantial diurnal and seasonal fluctuations.

The occurrence and strength of cold‐air pooling—and hence its influence on microclimate—is sensitive to climate change. Decreasing cloudiness may promote more frequent and intense cold‐air pooling due to enhanced night‐time cooling, while increasing cloud cover can suppress it. A recent process‐based analysis further suggests that greenhouse‐gas‐induced warming may weaken the nocturnal cold‐air drainage by at least 10% under a high‐emissions scenario (RCP 8.5), with the largest reductions expected in warm and humid regions (Rupp et al. 2021). Similarly, changes in wind speed and direction can either disrupt or enhance cold‐air pooling events depending on local exposure and terrain configuration (Miró et al. 2018). These interactions mean that cold‐air pooling‐prone areas, such as forested slopes and closed basins, may experience slower rates of warming compared to adjacent open sites (Daly et al. 2010; Pastore et al. 2022). Conversely, weakening inversions due to increased cloudiness or reduced snow cover (as cold air forms over snow patches) may accelerate warming (Figure 4b). In this way, shifts in the frequency, strength, and spatial distribution of temperature inversions can contribute to increasing the heterogeneity in microclimate warming rates across the landscape.

Current evidence for different rates of climate warming due to cold‐air pooling is mixed. Using 45 years of observational data from multiple meteorological stations in a forested mountain watershed, Jones et al. (2025) found little variation in multi‐decadal climate trends among sites, despite frequent occurrences of cold‐air pooling and temperature inversions. Hiebl and Schöner (2018), on the other hand, exploited a gridded observational dataset of daily minimum temperature across Austria over the 1961–2017 period and found that winter inversions had declined in frequency by 11%, leading to accelerated warming rates at low elevations. This illustrates the strong context dependency and therefore the need to better understand how changes in mesoclimatic conditions affect microclimatic changes over longer periods.

### 2.2 Clouds

Changes in cloud cover—including timing, altitude, and extent—strongly affect local radiation balances, thereby shaping microclimate change patterns. On clear summer days with high incoming solar radiation, temperature contrasts between adjacent microclimates peak due to spatial differences in shortwave radiation (e.g., Lehtinen et al. 2026). Low‐altitude clouds, like stratus and cumulus, block incoming solar radiation, thereby reducing spatial temperature contrasts near the surface. Observed and projected trends show that global average daytime cloud fraction (particularly low‐level cloudiness) decreases faster than night‐time cloud fraction (Luo et al. 2024b), but that there is a large variability across regions and seasons, as well as a high uncertainty in predictions due to cloud‐atmosphere feedbacks (Hou et al. 2021).

Reductions in daytime cloud cover such as those reported over Europe, where cloud cover has declined by roughly 1.5%–2.0% between 1980–1995 and 2000–2015 (Dong et al. 2022), increase the amount of sunlight reaching the surface (Sfîcă et al. 2021). This accelerates warming on sun‐exposed slopes compared to slowly‐warming pole‐facing slopes, increasing their contrast in near‐ground temperature (Chesnoiu et al. 2025; Figure 5; see also Case Study 1, Box 3, Figure 6e). The effect also occurs in moderately complex terrains at low sun angles, (Guthrie 2001), or across canopy cover gradients (Grimmond et al. 2000; Johnson and Smith 2008). The magnitude of this asymmetry depends on cloud thickness, sun angle, and surface albedo (e.g., from snow, soil, or vegetation).

**FIGURE 5:**
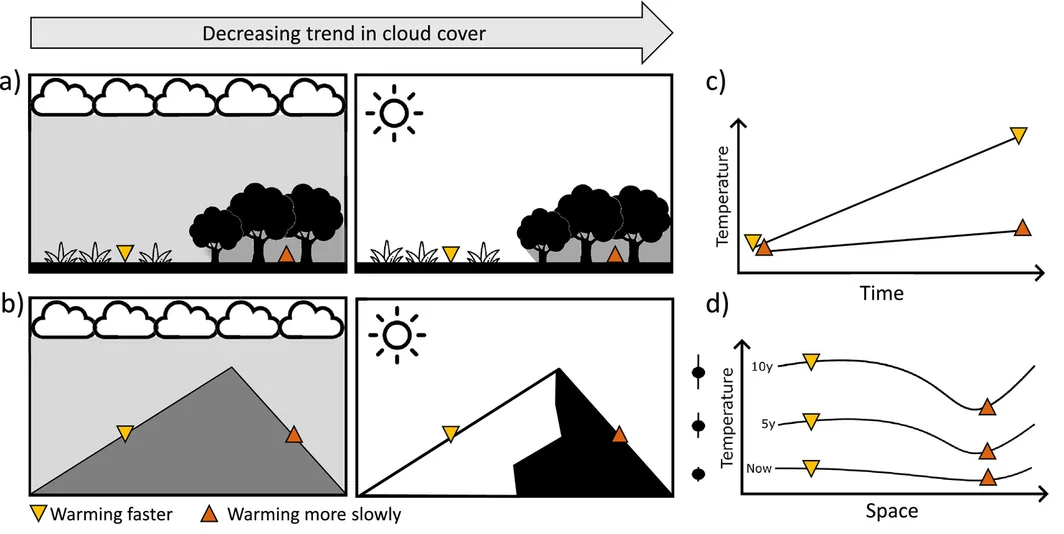
Reductions in daytime cloud cover cause nearby microclimates to diverge over time, increasing microclimate variance across space. With decreasing cloudiness, open meadows warm faster than forest interiors (a) and sun‐exposed slopes warm faster than shaded slopes (b). (c) Warming trajectories of adjacent microclimates with different warming trends (yellow vs. orange triangle, meadow vs. forest interior in a and sun‐exposed vs. shaded slope in b). (d) Visualizes a possible scenario where macroclimate temperature increases and cloudiness decreases over three time steps (now, +5 years, +10 years), thereby increasing microclimate contrasts over short distances (yellow vs. orange triangles).

**FIGURE 6:**
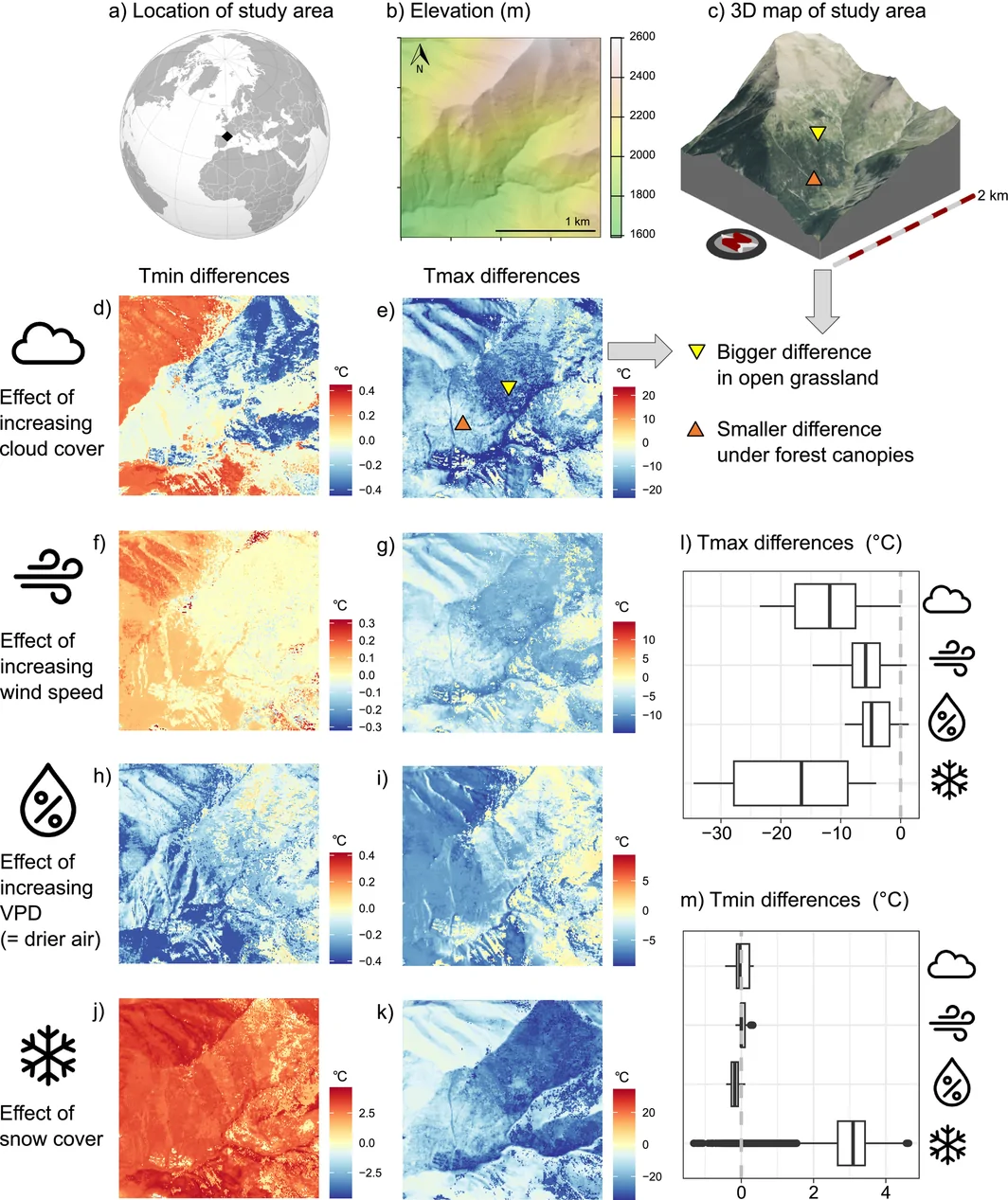
Model results for Case Study 1 in the Spanish Pyrenees showing the effects of changes in individual meteorological drivers on near‐surface temperature (5 cm above ground). Panels d, f, h and j show changes in minimum temperature, while panels e, g, i and k show changes in maximum temperature, resulting from experimentally altering cloud cover, wind speed, air humidity (expressed as vapor pressure deficit, VPD), and snow cover, respectively. These difference maps (d–k) show the temperature change obtained by comparing simulations with low and high values of each meteorological variable (halved versus doubled values), while holding all other variables constant, providing a measure of the sensitivity of microclimate to each driver across the landscape. Panels l and m summarize the distributions of temperature changes shown in the maps for maximum and minimum temperatures, respectively (see Table S1 for summary statistics). Small triangles indicate locations used to illustrate differences in the magnitude of vegetation‐mediated microclimate change. Map lines delineate study areas and do not necessarily depict accepted national boundaries (Icons from Flaticon.com).

Clouds also play a key role at night. While daytime temperature contrasts are largely driven by incoming solar radiation, night‐time cooling is governed by the loss of longwave radiation from the surface to the atmosphere. Clouds absorb this outgoing longwave radiation and—because they are efficient emitters of thermal radiation—emit part of it back toward the surface, reducing radiative cooling, inhibiting cold‐air formation and pooling in topographic depressions (Figure 5) and generally homogenizing temperatures across a landscape (Figure 6d). For example, Groot and Carlson (1996) found the difference of near‐ground night temperatures between open environments and under shelterwood to be larger in calm clear‐sky nights compared to nights with an overcast sky.

### 2.3 Precipitation

One facet of climate change is shifts in the amount, timing, frequency, and intensity of precipitation (IPCC 2023b) and, linked to that, the amount of moisture in the soil and humidity in the air. While the impact of changes in precipitation on soil moisture may depend strongly on climate regime, topography, vegetation, and soil type, more precipitation generally leads to increases in soil moisture (Cowley et al. 2017; Litaor et al. 2008; Sehler et al. 2019). Soil moisture is an important driver of near‐ground air and soil temperatures, especially under warm conditions (Ashcroft and Gollan 2013; von Arx et al. 2013). When soils are dry, most of the incoming solar energy is converted into sensible heat, warming the soil surface and the overlying air, rather than being used for evaporation. Conversely, when soils are wet, a larger fraction of the available energy is converted into latent heat fluxes through evaporation. Soil moisture also changes how heat moves through the soil as well as how much heat a soil can store. Compared to dry soils, moderately moist soils conduct heat rapidly away from the surface, while fully saturated soils act as substantial energy stores due to water's high heat capacity. Both mechanisms effectively buffer near‐surface soil temperature fluctuations, though saturated conditions provide the strongest temperature buffering at the surface (Campbell and Norman 2012). Consequently, daytime near‐ground air temperatures are often lower and night‐time temperatures higher over wet soils than over dry soils (Davis et al. 2019; García‐García et al. 2023). These effects will, however, depend on the ecosystem's soil moisture levels since increasing precipitation will have larger effects on microclimate in drylands compared to more mesic habitats (Korell et al. 2021). Additionally, soil moisture has been shown to limit evapotranspirative cooling stronger in open ecosystems compared to forest ecosystems, which has caused daytime summer land surface temperature to increase faster in open grasslands and croplands and more slowly in forests with a difference of −0.19 °C/decade (Li et al. 2025).

The timing of rainfall can also be important for microclimatic conditions. For example, increased summer rainfall generally cools soils by increasing latent heat fluxes and heat capacity (Dai et al. 1999; Mengistu et al. 2017). In contrast, winter rainfall often has smaller and more variable effects on soil temperature. In regions where soils remain unfrozen, increased winter rainfall raises soil moisture, allowing heat to be transferred and stored more efficiently in the soil, which can result in warmer winter soils. Areas with pronounced rainfall seasonality may therefore experience stronger seasonal changes in microclimatic buffering than regions with year‐round precipitation. Future changes in precipitation variability remain uncertain, adding further uncertainty to predictions of microclimate change (van der Wiel and Bintanja 2021; Zhang, Ding, et al. 2021).

While changes in rainfall are often considered, other forms of precipitation also exhibit temporal trends and influence microclimatic conditions. For example, there is a global reduction in fog (Klemm and Lin 2016) and dew (Dou et al. 2021), though with regional exceptions (Lavigne and Liu 2022). Particularly in arid systems, regular fog events decrease evapotranspiration (thus latent heat fluxes) and drought risk (Baguskas et al. 2016), while also shaping microclimate conditions by attenuating solar radiation, increasing relative humidity, and buffering thermal extremes (Sotomayor and Drezner 2019). Fog buffers minimum temperatures (Zhang et al. 2014), and a decrease in fog would likely lead to higher microclimate variability, including lower minimum temperatures.

In cold environments, changes in the depth, timing and duration of snowpacks can have large impacts on microclimates, particularly immediately above and below the snow layer. Due to snow's low thermal conductivity, it forms a strongly insulating layer, decoupling soil and surface temperature from free atmospheric temperature (Aalto et al. 2018). Under snow cover, surface temperatures cannot exceed 0°C because surplus energy input is absorbed as latent heat during melting, effectively buffering the surface against further warming. Therefore, even a thin layer of snow effectively decouples near‐ground maximum temperatures from macroclimate maximum temperatures and homogenizes microclimates across a landscape (See also Case Study 1, Box 3, Figure 6k). Minimum temperatures (below freezing point) below the snow are closely linked to snowpack thickness, with greater snow depth increasing microclimatic buffering up to a threshold depth (often 15–40 cm; with more compact snow having a lower buffering capacity), beyond which additional snow has limited impact (Wilson et al. 2020; Zhang 2005). Snowpack surfaces are characterised by a higher albedo and, as a result, generally lower near‐surface maximum temperatures than adjacent snow‐free microsites, with this effect being strongest at high sun angles (Zhang 2005). During low sun angles, on the other hand, experiments show that snow removal reduces winter soil temperatures, as frost penetrates more easily into exposed grounds (Zhao et al. 2022; Figure 6j). During snowmelt, sites with thicker snowpack also extend the seasonal soil temperature stability, reducing near‐surface frost events, and maintaining higher soil moisture. Thus, the general trend for reduced snowfall and shorter snowpack duration (see, e.g., IPCC 2023b), is likely to alter microclimates year‐round.

### 2.4 Wind

Considerable changes in wind speed and direction have been observed in recent decades, with global patterns showing marked heterogeneity (McVicar et al. 2012). Terrestrial systems have generally experienced wind‐stilling (i.e., a decrease in mean wind speed), but trends and projections vary by region (Zha et al. 2021) and may reflect changes in large‐scale ocean–atmosphere circulation patterns, such as the North Atlantic Oscillation (Zeng et al. 2019). Wind affects the coupling between ambient and near‐surface temperature or humidity by changing the atmospheric mixing close to the ground. Stronger winds enhance convection (see Box 2) as well as vertical and horizontal air mixing, leading to reduced microclimatic heterogeneity and a tighter coupling of micro‐ to macroclimate fluctuations (Ashcroft et al. 2009; Baker et al. 2021; Lehtinen et al. 2026; Figures 6f,g and S1g–i). However, this wind‐induced homogenization is reduced in forested and topographically complex landscapes, where terrain sheltering and surface roughness limit air mixing (Renaud et al. 2011; Wever 2012). Thus, increased windiness is expected to reduce microclimate variability most strongly in flat and open landscapes. In our model scenario, increased wind speed generally cooled maximum temperatures (in agreement with, e.g., Riddell and Porter 2025), but this cooling effect was weaker under forest canopies compared to open grasslands (Figures 6g and S2g–i). In contrast, windstilling is expected to increase microclimatic extremes by reducing atmospheric mixing, leading to more frequent cold air pooling (Dobrowski 2011) and more extreme thermal minima (Geiger 1966). Under windstilling, reduced atmospheric mixing also leads to thicker leaf/surface boundary layers (i.e., the thin layer of air directly adjacent to a surface), decreasing evaporation and likely increasing near surface humidity (Jones 2014).

Shifts in wind direction (McInnes et al. 2011) may lead to a spatial redistribution of wind‐sheltered and wind‐exposed microsites, with microsites that become more sheltered from wind potentially experiencing, for example, more extreme temperatures, greater dew accumulation and lower evaporation (Ashcroft et al. 2009; Geiger 1966; Maclean et al. 2017). Climate models show a large regional variability in projections in annual average and seasonal wind speeds (McInnes et al. 2011), and if any unidirectional long‐term trends emerge at all, they are likely very region‐specific.

BOX 3 **Case Study 1 on effects of meteorological conditions.**

Here, we present the model results for Case Study 1 showing minimum (Figure 6d,f,h,j,m) and maximum (Figure 6e,g,i,k,l) near‐surface temperatures 5 cm above ground. A selection of individual meteorological variables (cloud cover, wind speed, VPD, and snow cover) were modified independently to visualize their influence on microclimate temperatures and the spatial variability of these effects across a ca. 2 × 2 km^2^ landscape at the border of Parque Natural Posets‐Maladeta in the Spanish Pyrenees (see Supporting Information A for modeling details). For each simulation, we modelled the minimum temperature of the 10 coldest days and the maximum temperature of the 10 warmest days per year by setting the focal meteorological variable to low and high values (with values respectively halved or doubled) while keeping all other variables constant. The simulations revealed substantial differences in the magnitude of microclimate responses among atmospheric drivers and between minimum and maximum temperature metrics (Figures S1, S2, and 6, Table S1). Although the outputs of a mechanistic model that integrates multiple complex and non‐linear processes are not always straightforward to interpret, our objective was primarily to illustrate that changes in atmospheric conditions can produce highly heterogeneous microclimate responses across the landscape. This spatial heterogeneity is evident from the patchy temperature difference maps (Figure 6d–k), which demonstrate that identical changes in meteorological forcing do not translate into uniform microclimate temperature changes across space. As mentioned earlier, we ran the simulations in a way to isolate the effect of single atmospheric variables, which in reality do not change in isolation, sometimes complexifying model interpretation. Moreover, because the *microclimf* model does not account for mesoclimatic processes such as cold‐air pooling, the spatial complexity of temperature patterns and trends in real‐world landscapes is likely even greater than suggested by our simulations.

1 **Minimum Temperature**

Average changes in minimum temperature were generally below 0.2°C, except for the effect of snow (Figure 6l,m, Table S1). With low cloud cover, heterogeneity in minimum temperatures was created among others by dense vegetation, which prevented the ground from cooling down as it captured emitted thermal radiation from the ground and gradually released the heat stored during the day. Under high cloud cover, almost all upwelling emitted thermal radiation was emitted back downward irrespective of vegetation cover or terrain position (Figures 6d and S1d–f).

Higher wind speeds at night enhanced turbulent mixing, reducing the build‐up of cold air near the surface and thereby raising minimum temperatures, particularly where wind speeds increased the most (Figures 6f and S1g–i).

Increasing VPD (drier air) led to slightly lower minimum temperatures (on average −0.2°C, Table S1) because the dewpoint temperature dropped, meaning the air could cool down more before condensation began. When the dewpoint was reached, condensation released latent heat, which slowed further cooling. Thus, drier air typically allows temperatures to fall a little lower overnight, yet in heterogeneous ways across the landscape (Figures 6h and S1j–l).

The most pronounced effect on near‐ground minimum temperatures was caused by snow cover, which was almost by an order of magnitude larger than the effects of wind or air humidity/VPD with an average temperature change of 3°C (Figure 6j,m, Table S1). Snow cover had an insulating effect on near‐ground minimum temperatures, protecting the ground from frost and therefore leading to a general warming. However, the insulating effect depends on snow depth, density, moisture content, or soil heat flux and could therefore produce slight temperature gradients across the landscape (Figures 6j and S1m–o).

2 **Maximum Temperature**

Maximum temperature effects were overall larger compared to effects on minimum temperature. Spatial heterogeneity in maximum temperatures near the ground was higher under low cloud cover as the topographic and vegetation shading effects became more pronounced. When cloud cover increased, maximum temperatures generally decreased due to reduced energy input from the sun (on average by −12.4°C, Table S1), and this cooling effect was strongest in open environments (Figures 6e and S2d–f).

Wind had a cooling effect on maximum temperatures (on average −5.6°C, Table S1) as it increased turbulent mixing and transported heat away from the surface. This cooling effect was particularly strong on the open south‐east facing slopes—exposed to the mainly southerly winds in this area during the day—and less so under forest canopies which sheltered the ground from wind (Figures 6g and S2g–i).

Increasing VPD (drier air) had a cooling effect on maximum temperatures (on average −4.2°C, Table S1), as there was more evaporative cooling near the surface. The absolute effect varied throughout the landscape as a function of slope and aspect (which drive incoming energy), soil moisture, and vegetation height and density (Figures 6i and S2j–l).

When snow was present, maximum temperatures were effectively capped at around 0°C because additional energy input was used for melting the snow (latent heat of fusion) rather than further warming (Figures 6k and S2m–o). Depending on the location in the landscape, this resulted in a cooling effect ranging from −34.6°C to −4.1°C (Table S1).

## 3 PART II: Changes in Vegetation

Vegetation strongly influences microclimate buffering through its structural and physiological characteristics, including canopy height, Leaf Area Index (LAI) and stomatal conductance, that is, how easily water vapor passes through the stomata on leaf surfaces (De Frenne et al. 2021). Here we first consider physiological and phenological responses while community composition remains constant, before discussing structural and compositional changes.

### 3.1 Functional Responses

#### 3.1.1 Physiology

Without changing community composition, climate change can alter vegetation physiology, thereby modifying sub‐canopy microclimates. In temperature‐limited systems such as boreal forests and other high‐latitude or high‐altitude ecosystems, moderate warming can enhance primary productivity (Piao, Wang, et al. 2019). Rising atmospheric CO_2_ concentrations may further promote plant growth (Dong et al. 2025; Forkel et al. 2016; Mndela et al. 2022; Zhan et al. 2024), with elevated‐CO_2_ experiments reporting above‐ground biomass gains in forests of 21.4% on average (Baig et al. 2015). Nevertheless, this “carbon fertilization effect” is often constrained by nutrient and water availability (Ellsworth et al. 2017; Peñuelas et al. 2017; Terrer et al. 2019). Elevated atmospheric CO_2_ is expected to reduce stomatal conductance, limiting transpiration and increasing leaf and canopy temperatures, with a thermal effect of 1.3°C quantified in a mature oak canopy (Hagan Brown et al. 2025).

Climate change and the associated increase in climatic extremes can trigger rapid physiological responses, including thermal acclimation and tighter stomatal regulation (Bertolino et al. 2019; Liu et al. 2024; Wahid et al. 2007). These responses can help maintain plant function and canopy cover during heat stress, although reduced stomatal conductance and transpiration may simultaneously limit evaporative cooling and weaken microclimate buffering (Fernández et al. 2017; Wright and Francia 2024). Under severe heat or drought stress, reduced soil water availability and stomatal closure can increase canopy temperatures and weaken evaporative cooling (Grossiord et al. 2020; Slot et al. 2024). Once critical temperature thresholds are surpassed, leaf mortality and crown dieback can occur, causing a partial or complete breakdown of microclimate buffering (Doughty et al. 2023; Kopáček et al. 2020; Jump et al. 2017; von Arx et al. 2013). Nonetheless, recent studies have shown that elevated transpiration rates can persist during heatwaves even when carbon uptake ceases, under both well‐watered and drought‐stressed conditions (Marchin et al. 2023; Posch et al. 2024). This decoupling between transpiration and photosynthesis may allow thermal regulation during heat stress. Over longer time scales, enhanced productivity may also increase vegetation height, canopy density and LAI, linking physiological responses to the structural changes discussed below.

#### 3.1.2 Phenology

On interannual time scales, climate change has been shown to alter phenological cycles including leaf onset, flowering time and leaf senescence across a wide range of species and ecosystems (Badeck et al. 2004; e.g., Inouye 2022; Piao, Liu, et al. 2019). Warmer winters and springs generally advance leaf onset (Cai et al. 2025; Piao, Liu, et al. 2019), thereby reducing the rate at which spring microclimates change relative to macroclimate. In a mountainous region of Oregon (USA), years characterized by conditions resembling projected future climates—reduced snowpack and more frequent high‐pressure systems—not only advanced spring phenology but also increased phenological synchrony across elevation gradients, thereby reducing microclimatic heterogeneity (Ward et al. 2018). Autumn responses are more variable: warmer autumns delay senescence, whereas drought often accelerates it (Wu et al. 2022; Zhang et al. 2025; Zohner et al. 2023). Consequently, growing‐season responses to climate change vary among ecosystems. For example, substantial warming across China between 1960 and 2009 led to earlier leaf emergence by 5.4 days and delayed autumn leaf coloring by 4.6 days in four woody plant species, resulting in an overall growing‐season extension of approximately 10 days (Dai et al. 2014). In our Case Study 2, we showed how earlier leaf emergence in spring increased minimum temperatures and decreased maximum temperatures, mainly through changes in radiation: increased canopy cover reduced incoming shortwave radiation during the day and reduced night‐time longwave radiative heat loss (Box 4, Figure 7).

**FIGURE 7:**
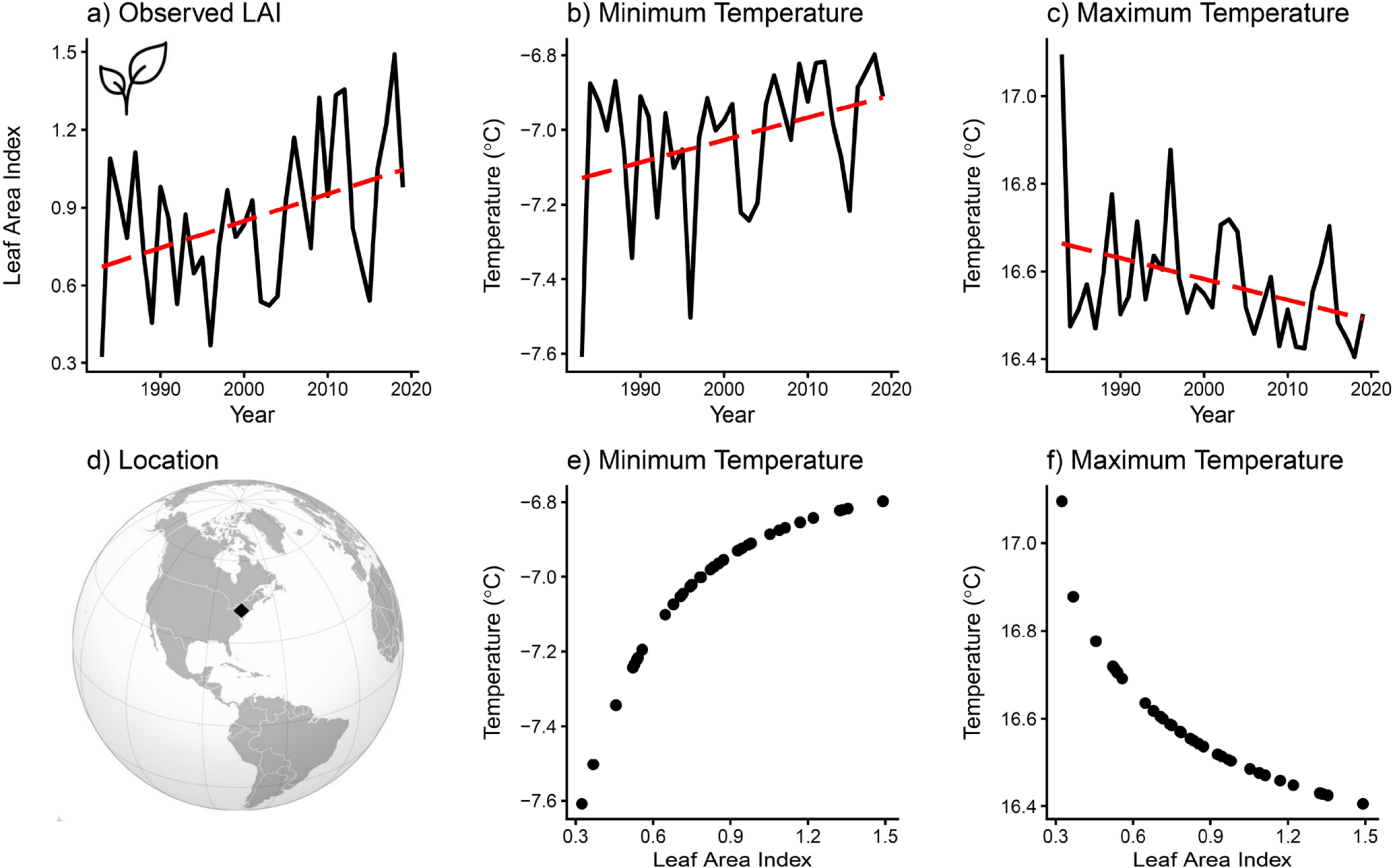
The influence of changes in leaf phenology on microclimate change in a mixed forest in New Hampshire, USA (Case Study 2). Observed mean monthly values of the leaf area index (LAI) in April between 1982 and 2019 (a) were used as an input to model forest understory minimum (b, e) and maximum temperature (c, f), all else—including macroclimate—being held constant. Map lines delineate study areas and do not necessarily depict accepted national boundaries.

Vegetation phenology can also feed back to the climate system through biogeophysical mechanisms such as changes in evapotranspiration, albedo and cloud formation (see section III). In a recent synthesis, Winkler et al. (2026) showed that a 10‐day extension of the growing season could lead to a global surface cooling of 0.10°C ± 0.03°C through changes in surface albedo and energy balance. However, the same study highlighted that increased spring transpiration and evaporative cooling may subsequently enhance summer soil drying, potentially offsetting some of this cooling effect through increased sensible heat fluxes.

### 3.2 Structural and Compositional Changes

#### 3.2.1 Vegetation Structure

Structural changes in vegetation can arise both through physiological responses within existing communities and through shifts in species composition. Increased productivity under warming and elevated CO_2_ may gradually produce taller and denser canopies with higher LAI, potentially increasing microclimate buffering, that is, warming night‐time minimum and cooling daytime maximum temperatures. Because this relationship is non‐linear, buffering changes most strongly at low LAI (Case Study 2, Box 4; Zellweger et al. 2019). Changes in canopy height and density also modify air movement and turbulent exchange, thereby influencing the transfer of heat and moisture between the vegetation and the atmosphere. In microclimate models, these effects can be represented through aerodynamic properties of the canopy.

A well‐documented example of vegetation‐mediated microclimate change is the “shrubification”, that is, the expansion of woody species into previously treeless ecosystems, such as tundra (Sturm et al. 2001) or savannas (Devine et al. 2017; Case Study 3, Box 4). Shrub expansion increases vegetation height, leaf area, transpiration and shading, buffering daytime temperatures while often increasing winter soil temperatures through snow insulation (Finne et al. 2023; Kemppinen, Niittynen, Virkkala, et al. 2021; Kropp et al. 2021; Mekonnen et al. 2021; Myers‐Smith and Hik 2013; Wilcox et al. 2024). More generally, plant species influence microclimatic conditions through their structural characteristics, which can be partly described by traits such as canopy height, cover, and shade‐casting ability (Gril, Spicher, et al. 2023; Meeussen et al. 2021). Microclimatic effects are strongest when replacement species differ markedly in stature or phenology, for example, when shifts occur between grasslands to shrublands or from evergreen to deciduous dominance. For instance, in low‐growing shrublands, non‐native trees lowered near‐surface air temperatures by increasing shading, whereas dense invasions raised near‐surface temperatures by suppressing native ground cover and exposing more bare ground (Garcia and Clusella‐Trullas 2019). Furthermore, forest disturbances such as droughts, pest insects, fires and storms reduce canopy buffering and cause rapid increases in daytime summer sub‐canopy temperature (e.g., Greiser et al. 2025). They can effectively erase decades of thermal decoupling, exposing the understory to thermal conditions equivalent to several decades of macroclimate warming (Haesen et al. 2026). Increasing structural diversity can have the opposite effect. In a large‐scale tree diversity experiment in China, near‐surface temperatures were up to 4.4°C lower in 24‐species mixtures than in monocultures through greater canopy density and structural complexity (Schnabel et al. 2025). Likewise, increased species diversity can enhance canopy density and rooting depth diversity, leading to higher evapotranspiration rates and stronger microclimate buffering (Huang et al. 2024; Schnabel et al. 2025; Zhang et al. 2022).

#### 3.2.2 Surface Albedo

Species favored by climate change can differ in albedo, thereby altering the amount of solar radiation absorbed at the surface. For instance, in boreal forests, birch exhibits a higher stand albedo (0.22 ± 0.01) than Scots pine (0.14 ± 0.02) and Norway spruce (0.11 ± 0.03, Lukeš et al. 2013). Arctic and alpine greening has emerged as a major trend at high latitudes and elevations, driven by warming and a lengthened growing season (Choler et al. 2021; Roland et al. 2024; Rumpf et al. 2022). In these regions, open land is typically snow‐covered in winter or lichen‐dominated in summer, both characterized by high albedo. However, shrub‐dominated communities can have half the albedo values of the lichen *Cladonia stellaris* in mountainous Norway (Aartsma et al. 2020), thereby enhancing microclimate warming through increased absorption of solar radiation (Mallen‐Cooper et al. 2021). Comparing satellite‐derived land surface temperature trends between forests and open lands in Europe, Li et al. (2025) demonstrated that decreases in winter snow cover decreased the albedo differences and thereby the temperature differences between forested and open habitats, with open grasslands warming −0.14 °C/decade faster than forests. Conversely, in warmer regions, rising temperatures and declining precipitation may promote desertification, where reduced vegetation cover increases surface albedo (IPCC 2022).

#### 3.2.3 Transpiration

Community turnover also alters transpiration through changes in species composition and functional traits. Many species replacing current species assemblages possess morphological and hydraulic traits that enhance their ability to cope with new climatic conditions (Kramp et al. 2022; Kühn et al. 2021). In some alpine meadows, colonizing lowland species exhibit larger leaves (Debouk et al. 2015; Schuchardt et al. 2023), which increase transpiration rates, elevate air humidity, and reduce temperatures through latent heat exchange—though only in ecosystems where water availability is not limiting. In regions facing more frequent droughts, however, “winner” species are likely to be characterized by short stature, sparse canopies, small and thick leaves, and tighter stomatal regulation. The increasing dominance of such water‐conserving species may weaken vegetation‐mediated cooling (Michalet et al. 2024; Richter et al. 2022). Deep‐rooted drought‐adapted species may better maintain canopy cover during prolonged drought (Kühn et al. 2021).

BOX 4 **Case studies on vegetation effects.**

**Case Study 2: Long‐term shifts in leaf phenology**

To assess how variation in leaf phenology influences microclimate buffering, we simulated near‐surface air temperature beneath a forest canopy in a mixed, predominantly deciduous forest in New Hampshire, USA. To isolate the effects of phenology, the observed mean monthly values of the leaf area index (LAI) in April between 1982 and 2019 were used as an input to model forest understory monthly minimum and maximum temperature, all else—including macroclimate—being held constant (see Supporting Information A for modeling details). Between 1982 and 2019, the study forest observed an average increase in April LAI of 0.104 per decade from 0.679 to 1.054 (Figure 7a), raising modelled minimum temperature from −7.1°C to −6.9°C (Figure 7b) and reducing maximum temperature from 16.7°C to 16.5°C (Figure 7c), with strongest changes at low LAI (Figure 7e,f). Earlier leaf emergence increased buffering by altering both shortwave and longwave radiation, reducing daytime warming and night‐time cooling.

**Case Study 3: Shrub and tree encroachment**

We modeled the effect of increased tree and shrub cover on minimum and maximum near‐ground air temperature in a 1 × 1 km^2^ grassland area in the Central Anatolian Steppe of Niğde Province, Western Turkey. Invasion of shrubs and trees was modeled by superimposing structural vegetation data, namely vegetation height (Figure 8a,b) and LAI, from a nearby steppe‐woodland site onto a digital terrain model of the grassland site. Minimum and maximum temperatures (at 5 cm above ground) refer to the average of daily minimum and maximum temperatures of the years' 10 coldest or hottest days, respectively (Figure 8e,f,h,i).

**FIGURE 8:**
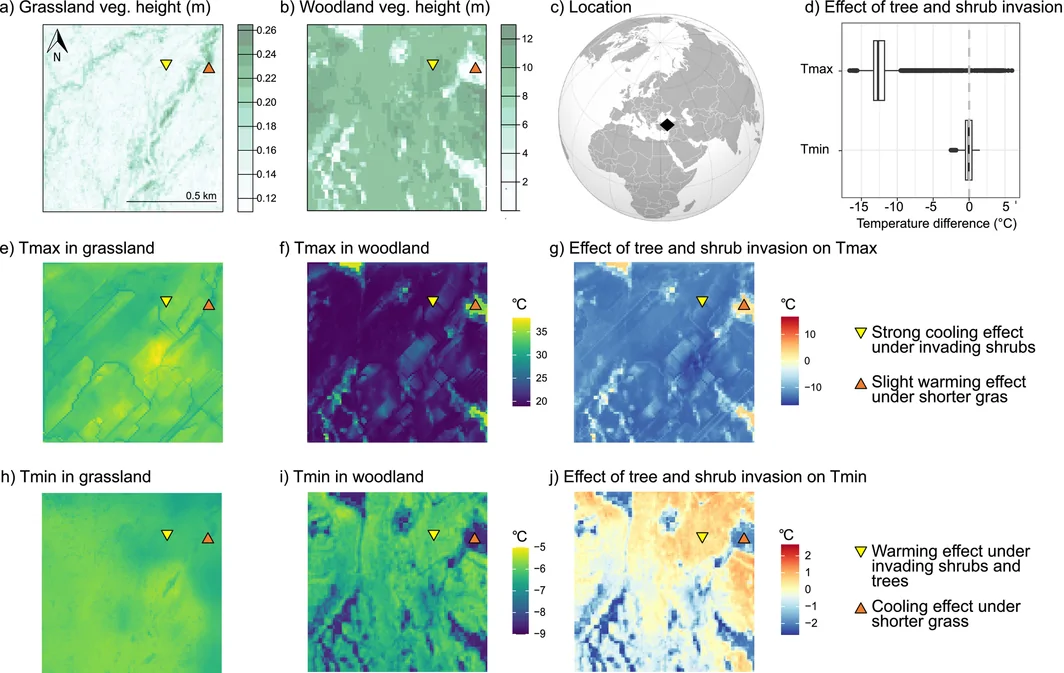
The effect of increased tree and shrub cover on minimum and maximum near‐ground air temperature in a grassland area in the Central Anatolian Steppe of Niğde Province, Western Turkey (Case Study 3). (e, f, h, i) Minimum and maximum temperatures refer to average of daily minimum and maximum temperatures of the years' 10 coldest or hottest days, respectively. (g, j) Temperature difference between the grassland and the woodland scenario, negative (blue) values mean temperatures are getting cooler. The difference (microclimate change) maps are summarized in boxplots (d). Both the average magnitude and the spread of microclimate change are larger for maximum than for minimum temperatures (d, see also Table S2 for summary statistics). The small triangles mark example locations with different magnitudes of microclimate change caused by different trends in vegetation cover. Map lines delineate study areas and do not necessarily depict accepted national boundaries.

The modelled change in vegetation cover generally led to warmer near‐ground minimum temperatures (on average by 0.2°C) and cooler maximum temperatures (on average by 11.9°C, Table S2). However, microclimate change varied across the study area as a function of vegetation height and leaf area index (Figure 8g,j). For example, we observed changes of maximum temperature ranging from −16.6°C (cooling) under tall dense shrubs and trees to +6.0°C (warming) in open patches where grass height was modelled to become shorter (Table S2, Figure 8d,g). Minimum temperature changes varied less than maximum temperatures and ranged from −2.5°C under invading shrubs and trees to 1.5°C in patches with shorter grass (Table S2, Figure 8d,j). Similar to Case Study 2, these temperature changes mainly resulted from changes in the radiation balance at day and night.

## 4 PART III: Feedbacks Vegetation and Microclimate Feedbacks

The processes described in the previous sections not only determine how climate change alters microclimates but can also feed back to influence regional and global climate. At the global scale, climate feedbacks regulate the rate of warming driven by greenhouse gas emissions, whereas at local and regional scales, feedbacks arise through changes in surface energy exchange, hydrology, and vegetation that can influence the atmosphere. We therefore distinguish between global climate‐system feedbacks and local and regional land‐atmosphere feedbacks that originate at the land surface and are driven by land surface features including vegetation.

At global scales, the primary stabilizing mechanism of the climate system is the Planck feedback, whereby warming surfaces emit more thermal radiation (IPCC 2023a). This negative radiative feedback limits global warming, while local energy exchange processes (Box 2) determine how global warming is expressed in near‐surface microclimates. In contrast, warming increases atmospheric water vapor, strengthening the greenhouse effect through a positive feedback. Together with the closely coupled lapse‐rate feedback, this contributes approximately +1.3 W/m^2^/°C to the global climate feedback (IPCC 2023a). Clouds strongly influence Earth's radiation balance by reflecting incoming solar radiation while also trapping outgoing longwave radiation. The net cloud feedback remains uncertain and varies among cloud types, regions, and seasons (Sherwood et al. 2020). Changes in surface albedo provide another major feedback, with the loss of highly reflective surfaces such as snow and ice, or the expansion of darker vegetation, reinforcing warming (Bright et al. 2014; Evans et al. 2017; Jiao et al. 2017; Pearson et al. 2013; Pecl et al. 2017).

At local and regional scales, land–atmosphere exchanges provide an important pathway by which microclimates feed back to the atmosphere. Vegetation strongly regulates the exchange of energy and water between the land surface and atmosphere (De Frenne et al. 2021; Miralles et al. 2025; Winkler et al. 2026). Vegetation cools leaves and surrounding air through transpiration while increasing atmospheric water vapor, thereby potentially promoting cloud formation (Duveiller et al. 2021). Conversely, vegetation loss caused by drought, repeated heat stress or disturbance can accelerate surface warming (IPCC 2023a). Besides regulating radiation and evapotranspiration, vegetation structure also alters the aerodynamic roughness of the land surface. Taller and structurally more complex vegetation generally enhances turbulent exchanges of heat, moisture and momentum between the land and atmosphere, influencing boundary‐layer development, cloud formation and regional climate (Miralles et al. 2025). Vegetation can further influence regional cloud formation and precipitation through emissions of BVOCs and atmospheric pollen, which contribute to atmospheric aerosols (Dada et al. 2023; Duveiller et al. 2021; Greenberg et al. 2004; Kretzschmar et al. 2024; Rap et al. 2018). Longer‐term vegetation–climate feedbacks arise through shifts in vegetation structure and tree‐line position, which alter surface albedo, evapotranspiration and carbon storage. Trees and shrubs can facilitate their own persistence and expansion by locally elevating night‐time temperatures (D'Odorico et al. 2013), reinforcing these broader climate feedbacks. Deforestation in the Amazon has increased low‐altitude cloud cover, producing top‐of‐atmosphere cooling of up to 6.8 ± 0.6 W/m^2^ in areas of extensive forest loss (Dror and Feingold 2026), illustrating that the local climatic effects of deforestation and its top‐of‐atmosphere radiative effects do not necessarily act in the same direction.

Beyond these biophysical feedbacks, microclimates also influence climate through biogeochemical feedbacks associated with the carbon cycle (Arora et al. 2020; Song et al. 2019). While these processes are typically represented in coarse‐resolution Earth system models, many are influenced by microclimate. One major feedback mechanism is the release of long‐stored CO_2_ and methane from thawing permafrost, enhancing atmospheric greenhouse gas concentrations and adding an estimated 0.29°C ± 0.21°C of warming under RCP8.5 and 0.05°C–0.15°C under RCP4.5 (Schuur et al. 2022). Similarly, climate change may induce forest dieback, releasing CO_2_ into the atmosphere and reinforcing warming (Munday et al. 2025). Conversely, higher atmospheric CO_2_ concentrations can temporarily enhance plant growth and carbon uptake (Norby 2025), although this fertilization effect is constrained over time by nutrient limitation, drought stress and shifts toward less carbon‐dense vegetation (Peñuelas et al. 2017; Terrer et al. 2019). Likewise, climate‐driven transitions from forests and shrublands to more open ecosystems reduce carbon storage and reinforce atmospheric CO_2_ increases (Rosan et al. 2019; Venter et al. 2018).

Together, these examples illustrate how local microclimatic processes can influence regional meteorology directly through energy and water exchange, and potentially global climate through vegetation‐mediated changes in energy, water, and carbon cycling. While the strongest and most consistent feedbacks operate through surface energy exchange, hydrological processes, albedo change, and carbon storage, many remain context dependent and poorly quantified. Key research priorities include quantifying the strength and timing of these feedbacks, integrating microclimatic processes into biosphere–atmosphere models, and resolving physiological limits, adaptation, time lags, non‐linear responses, and regime shifts in vegetation–climate feedbacks (Winkler et al. 2026).

## 5 Conclusions and Outlook

This review highlights three overarching conclusions. First, microclimate warming does not necessarily track macroclimate warming, and nearby locations may experience substantially different warming rates, leading to increased or decreased heterogeneity of microclimate temperatures (Figure 1). Second, these divergent warming trajectories and shifts in spatial heterogeneity arise from the combined effects of changing meteorological conditions, vegetation dynamics, and land‐atmosphere feedbacks rather than from a single dominant driver (Figure 2). Third, predicting future microclimates therefore requires moving beyond static microclimate‐macroclimate offsets and instead accounting for the physical and ecological processes governing microclimate change. Ultimately, this microclimatic perspective may improve predictions of ecological responses and help anticipate climate impacts at scales relevant to organisms and ecosystems.

Although this review focuses largely on trends in mean microclimate change, such averages may obscure ecologically critical shifts in the frequency, intensity, and duration of extreme events—often the primary triggers of biological responses like sudden range shifts (Soifer et al. 2025) or ecosystem tipping‐points (Lenton 2013). Importantly, microclimate changes can be spatially heterogeneous, and therefore what constitutes an extreme event at a regional scale (e.g., heat waves or droughts) may be locally amplified or buffered.

Microclimate ecology has recently focused on increasing the spatial resolution of climate datasets, but we argue that it is at least equally important to understand the processes driving their temporal dynamics and how these processes manifest across landscapes and ecosystems. A major unresolved challenge is quantifying turbulent transport of heat and moisture in structurally complex vegetation. This limits our ability to predict how effectively vegetation buffers or amplifies warming and how much structural complexity must be represented in climate models. These uncertainties propagate into our understanding of vegetation–microclimate feedbacks, because vegetation responses themselves depend on fine‐scale spatial and temporal variation in microclimate that is often poorly resolved, particularly under novel future climate conditions.

Resolving these uncertainties requires long‐term observations linking vegetation change and microclimate across heterogeneous landscapes. The growing availability of long‐term microclimate records and the increasing length of these time series (Lembrechts et al. 2020) hold great value for capturing fine‐scale spatiotemporal patterns (Haesen et al. 2026). Microclimate observation can also strategically prioritize data collection that supports the development, refinement, and validation of mechanistic models—such as those we used in this paper—building on available open‐source approaches (Buckley et al. 2023; Kearney and Porter 2017; Maclean 2026). Grounded in physical principles, mechanistic models explicitly represent underlying processes and can extrapolate beyond observed conditions, making them valuable tools for forecasting and hindcasting (Malmborg et al. 2024). However, these models require joint measurements of microclimate and vegetation for validation, which remain rare. Nevertheless, the microclimate community has made impressive progress in producing high‐resolution gridded products of microclimate temperature at local (Gril, Laslier, et al. 2023; Niittynen et al. 2024), regional (Brůna et al. 2026; Greiser et al. 2018), continental (Haesen, Lembrechts, et al. 2023), and global scales (Lembrechts et al. 2022). While these datasets offer broad spatial coverage, many represent static microclimate landscapes despite substantial temporal variability and uncertainty. Researchers should therefore critically evaluate their temporal representativeness and develop gridded products that better integrate spatial and temporal dynamics (Klinges et al. 2024).

Many of the physical principles governing microclimates have been established through decades of work in micrometeorology, boundary‐layer climatology, and land–atmosphere science (Bonan 2016, 2019; Oke 1987). However, these advances remain only partly integrated into ecological research (Klinges et al. 2026), while the representation of vegetation, and the complexity of the biosphere, in many atmospheric and land‐surface models is still largely based on a few broad plant functional types that inadequately capture species composition, vegetation dynamics, disturbance, and fine‐scale variation in vegetation structure. Progress will require closer integration among ecology, plant ecophysiology, and atmospheric sciences. Ecologists increasingly need to move beyond treating microclimates as static offsets from macroclimate and engage with turbulent exchange, canopy coupling, and land–atmosphere interactions, while also seeking collaborations with atmospheric scientists when developing or applying microclimate models and interpreting field observations. Atmospheric scientists, in turn, need to better represent vegetation dynamics and biologically relevant microclimatic heterogeneity (Kemppinen et al. 2024). Plant ecophysiology provides a key bridge because vegetation both modifies and responds to microclimate through transpiration, phenology, growth, and stress responses. While many of the underlying physical principles are well established, integrating them with ecological and ecophysiological understanding remains a major interdisciplinary challenge. We intend this review to serve as a bridge between ecology and atmospheric science by providing an accessible synthesis of the physical processes governing microclimate change while highlighting the need for closer collaboration between these disciplines. Much of the foundational knowledge needed for this integration has existed for decades (Geiger 1966)—what is now needed is the interdisciplinary will to apply it.

## Author Contributions

**C. Greiser:** conceptualization, supervision, visualization, writing – original draft, writing – review and editing. **E. Gril:** conceptualization, supervision, visualization, writing – original draft, writing – review and editing. **I. M. D. Maclean:** conceptualization, data curation, formal analysis, supervision, visualization, writing – original draft, writing – review and editing. **R. Beugnon:** conceptualization, visualization, writing – original draft, writing – review and editing. **P. le Roux:** conceptualization, writing – original draft, writing – review and editing. **J. Kemppinen:** conceptualization, writing – original draft, writing – review and editing. **M. W. Rhodes:** conceptualization, data curation, formal analysis, visualization, writing – original draft, writing – review and editing. **J. Aalto:** conceptualization, writing – original draft, writing – review and editing. **D. M. Christiansen:** conceptualization, writing – original draft, writing – review and editing. **P. De Frenne:** conceptualization, writing – original draft, writing – review and editing. **S. Haesen:** conceptualization, writing – original draft, writing – review and editing. **D. Klinges:** conceptualization, writing – original draft, writing – review and editing. **M. Kopecký:** conceptualization, writing – original draft, writing – review and editing. **J. Lenoir:** conceptualization, writing – original draft, writing – review and editing. **M. Luoto:** conceptualization, writing – original draft, writing – review and editing. **M. Rantanen:** conceptualization, writing – original draft, writing – review and editing. **G. Strandberg:** conceptualization, writing – original draft, writing – review and editing. **L. van den Brink:** conceptualization, writing – original draft, writing – review and editing. **L. M. Mercado:** conceptualization, writing – original draft, writing – review and editing. **J. Lembrechts:** conceptualization, funding acquisition, supervision, writing – original draft, writing – review and editing. **K. Van Meerbeek:** conceptualization, funding acquisition, supervision, visualization, writing – original draft, writing – review and editing.

## Funding

This work was supported by Novo Nordisk Fonden (NNF22OC0078703), Deutsche Forschungsgemeinschaft (202548816 and Ei862/29‐1), Agence Nationale de la Recherche (ANR‐21‐CE32‐0012‐03, ANR‐24‐PEFO‐0003), Fonds Wetenschappelijk Onderzoek (W004024), Natural Environment Research Council (NE/V000772/1, NE/W004895/1, NE/W006618/1, NE/X001172/1), Research Council of Finland (342890, 353218, 370245), Grantová Agentura České Republiky (GACR 23‐06614S), European Fund for Regional Economic Development (CPER ECRIN), Region Bourgogne Franche‐Comté (ATRACT), France 2030 (A2ForBois), Région Hauts‐de‐France (CPER ECRIN), Horizon Europe European Research Council (101124948), Akademie Věd České Republiky (RVO 67985939), Svenska Forskningsrådet Formas (2021‐01993), and Ministère de l'Enseignement supérieur et de la Recherche (CPER ECRIN).

## Conflicts of Interest

The authors declare no conflicts of interest.

## Supporting information


**Figure S1:** Model results of Case Study 1 for minimum near‐surface temperature (5 cm above ground), where single meteorological variables (clouds, wind speed, air humidity, and snow cover) were modified in isolation to visualize their effect on microclimate warming and how it varies across a ca. 2 × 2 km^2^ landscape on the border of Parque Natural Posets Maladeta in the Spanish Pyrenees (See Supporting Information for more modeling details and an interpretation of observed patterns). Left and middle columns show the minimum temperature of the 10 coldest days per year when setting the respective meteorological variables at low and high values, respectively, and keeping all other variables constant. Difference maps (right column) show the differences between high and low simulated values of the meteorological variables, that is, the effect of going from low to high values (Icons from Flaticon.com).
**Figure S2:** Model results of Case Study 1 for maximum near‐surface temperature (5 cm above ground), where single meteorological variables (clouds, wind speed, air humidity, and snow cover) were modified in isolation to visualize their effect on microclimate warming and how it varies across a ca. 2 × 2 km^2^ landscape on the border of Parque Natural Posets Maladeta in the Spanish Pyrenees (See Supporting Information for more modeling details and an interpretation of observed patterns). Left and middle columns show the maximum temperature of the 10 warmest days per year when setting the respective meteorological variables at low and high values, respectively, and keeping all other variables constant. Difference maps (right column) show the differences between high and low simulated values of the meteorological variables, that is, the effect of going from low to high values (Icons from Flaticon.com).
**Table S1:** Summary statistics of the microclimate change induced by changing atmospheric variables (cloudiness, wind speed, VPD, and snow cover). IQR, interquartile range; min/max, absolute minimum/maximum; q1/q3, first/third quartile; SD, standard deviation.
**Table S2:** Summary statistics of the microclimate change induced by changing vegetation cover from grassland to shrub/tree dominated woodland. IQR, interquartile range; min/max, absolute minimum/maximum; q1/q3, first/third quartile; SD, standard deviation.

## Data Availability

The environmental data used in the illustrative modeling scenarios are publicly available from the sources described in the Supporting Information. Simulations were run using the R packages *microclimf* v2.0.1 (https://doi.org/10.5281/zenodo.18274614) and *micropoint* (https://github.com/ilyamaclean/micropoint, using Git commit c9d95d852fb576313165792e0ffda5c70e546361).
