# Supplementary material for: Climate Change Experienced by Life on Earth—Drivers and Mechanisms of Future Trends in Microclimates
Source: Glob Chang Biol. 2026 Oct 21;32(10):e71140. doi: 10.1111/gcb.71140 (PMC13685058; doi:10.1111/gcb.71140)
Supplement: Supplementary file 1 [file GCB-32-e71140-s001.pdf]

Supplementary Material

A - Modelling details

Greiser et al. (2026) - Climate change experienced by life on Earth - drivers and mechanisms of future trends in microclimates

Materials and methods

Study site selection

The selection of sites for the three case studies was guided by three considerations. First, the full author team evaluated which of the processes discussed in the manuscript could be quantified using the *microclimf* environment (Maclean 2026). Second, we identified regions that combined heterogeneity in relevant gradients of vegetation cover and topography with the availability of high-quality, open-access data on topography, vegetation, and weather or climate. Finally, we sought to distribute the three study sites geographically so that the case studies were not restricted to Europe. Once a region for each case study had been chosen, individual sites were selected for vegetation and topographic characteristics that would best illustrate the intended microclimate phenomenon.

Case study 1: Effects of changing weather on microclimate temperature

To estimate how changing weather affects spatial variability in microclimate warming, we simulated near-surface air temperature using the R package *microclimf* (Maclean 2026) across a 1990 × 1990 m² area on the border of Parque Natural Posets Maladeta in the Spanish Pyrenees (42.61°N, 0.37°E). The site spans subalpine and alpine terrain typical of the central Pyrenees, with elevations between 1590vm and 2602 m and a climate strongly influenced by both Atlantic and Mediterranean systems (García-Ruiz et al., 2020).

We used the *microclimf* package to model air temperature 5 cm above ground at hourly intervals and at a grid resolution of 10 m. Microclimf is a mechanistic microclimate model, written for the R programming environment that, for a set of user inputs provides a time-series of hourly estimates of microclimate above or within vegetated surfaces or below the ground using a set of standard equations derived using Monin-Obukhov Similarity (Foken, 2006) and Raupach's (1989) Localized Near-field theory model. Using this approach, microclimatic conditions are determined from local energy balance and landscape features, such as terrain and vegetation, are assumed to affect components of the energy balance. The model requires as inputs a set of hourly weather variables and gridded datasets of soil and vegetation properties and terrain.

To drive the model, hourly weather data for 2021 were obtained from the ERA5 reanalysis (Hersbach et al., 2020), produced by the European Centre for Medium-Range Weather Forecasts (ECMWF). A digital terrain model and vegetation height data were sourced from the Instituto Geográfico Nacional using datasets derived from the second lidar coverage flights (2015–2021) of the National Plan for Aerial Orthophotography. We used the Digital Terrain Model second coverage with 2 m grid spacing (MDT02-ETRS89-HU31-0179-2-COB2) and the 2.5 m spatial resolution Vegetation Digital Surfaces Model - nDSM2,5 2nd Coverage (NDSM-VEGETACION-H31-0179-COB2), respectively, with both datasets aggregated by the mean to 10 m spatial resolution. Leaf area index data were obtained for 9th July 2021 from the Copernicus Leaf Area Index dataset (2016–present, 10 m grid resolution, daily). All other vegetation inputs were derived from habitat type using the 'vegpfromhab' function included with the *microclimf* package.

For all scenarios, we identified the ten days with the 10 hottest and coldest hours of 2021 from hourly ERA5 air temperature. To assess the influence of changes in cloud cover, wind speed, and humidity, each was modified in isolation. Cloud cover is not a direct input to the model and changes were therefore simulated by adjusting downward longwave and shortwave radiation. For each of four

sets of variables, values were either halved (*low scenario*) or doubled (*high scenario*) while all other variables were held constant. To simulate the effects of snow, the model was run with the snow model turned on and off. For each scenario, *microclimf* simulations were re-run and summarised as (i) the mean of the daily maximum and minimum temperatures across the selected days, and (ii) the temperature difference between the means of the high and low scenarios. This allowed us to quantify the sensitivity of modelled near-surface microclimate to changes in each weather variable independently.

Case study 2: Effects of leaf phenology on trends in below-canopy microclimate

To assess how variation in leaf phenology influences microclimate buffering, we simulated near-surface air temperature beneath a forest canopy using the R package *micropoint* (https://github.com/ilyamaclean/micropoint). The study site was a mixed, predominantly deciduous forest in New Hampshire, USA (43.815°N, 72.038°W).

Monthly leaf area index (LAI) data for the site between 1982 and 2019 were obtained from the AVHRR satellite record (NOAA, 2020). To isolate the effects of phenology, we focused on April 2019 and repeatedly ran the *micropoint* model (Maclean 2026) using the same hourly weather data for that month, while substituting LAI with its April value from different years in the AVHRR record. Vegetation height data for the model were obtained from Global Canopy Height 2020 dataset held by Google Earth (Lang et al., 2023) using the "vegheight_dowland" function in *microclimdata* package (Maclean 2026), while all other vegetation parameters were set to the model defaults for deciduous forest.

Micropoint is, in effect, a point version of the *microclimf* grid model and is therefore a mechanistic, below-canopy microclimate model that estimates hourly temperature at user-defined heights beneath forest canopies based on canopy structure and energy balance processes. In each run, air temperature was simulated at 5 cm above the ground surface. Outputs were summarised as (i) daily minimum and (ii) daily maximum temperatures under each substituted LAI value. This design allowed us to quantify how interannual variation in leaf phenology affects near-ground microclimate conditions, independent of weather variability.

Case study 3: Effects of shrub and tree encroachment on steppe microclimate

We examined the influence of shrubification (heterogeneous invasion of shrubs and trees) on near-surface microclimate in the Central Anatolian Steppe of Niğde Province, western Turkey (37.97°N, 34.41°E). Simulations were conducted over a 1 × 1 km area using the R package *microclimf* (Maclean, 2026). Hourly weather data for 2020 were obtained from the ERA5 reanalysis (Hersbach et al., 2020). Similar to case study 1, we identified the ten days with the 10 hottest and coldest hours of 2021 from hourly ERA5 air temperature. For each day, *microclimf* was used to model hourly air temperature at 5 cm above the ground surface and 10 m spatial grid resolution.

Two vegetation scenarios were compared. The first represented the current grassland state, while the second represented a woodland state. The latter was constructed by superimposing structural vegetation data (e.g., canopy height, leaf area index) from a nearby woodland site (38.07°N, 34.23°E) onto a digital terrain model of the grassland site.

Ten-meter spatial resolution digital elevation data were sourced from the Amazon Web Services (AWS) using the 'dem_download' function included with the *microclimdata* package (Maclean 2026). For the shrub scenario, 10 m spatial resolution vegetation height data were obtained from the European Space Agency (ESA) using the "vegheight_downland" function in the same package. As this dataset is relatively low precision, however, it is unsuitable for differentiating small variations in grass height. For the grassland scenario, therefore, we derived estimates for grass height using the *vegpforgrass* function in the *micropoint* package, which scales height to total leaf area using an allometric equation derived from field observations of grassland across Europe (supporting information in Maclean 2026). Leaf area index data for both scenarios were downloaded from the Copernicus Leaf Area Index 2016-present (raster 10 m), Europe, daily dataset for the 2nd July 2020

(VI_20200702T082609_S2B_T36SXH-010m_V101_LAI). All other vegetation inputs were estimated from habitat type using the 'vegpfromhab' function included with the *microclimf* package.

Outputs were summarised as the mean of daily maximum (for the 10 hottest days) and minimum (for the 10 coldest days) temperatures across the year. To quantify the effect of shrub encroachment, we calculated the difference in near-surface temperatures between the scrub and grassland scenarios.

References

Foken, T. (2006). 50 years of the Monin–obukhov similarity theory. *Boundary - Layer Meteorology*, *119*(3), 431–447.

Hersbach, H., Bell, B., Berrisford, P., Hirahara, S., Horányi, A., Muñoz-Sabater, J., Nicolas, J., Peubey, C., Radu, R., Schepers, D., Simmons, A., Soci, C., Abdalla, S., Abellan, X., Balsamo, G., Bechtold, P., Biavati, G., Bidlot, J., Bonavita, M., … Jean-Noël Thépaut. (2020). The ERA5 global reanalysis. *Quarterly Journal of the Royal Meteorological Society. Royal Meteorological Society (Great Britain)*, *146*(730), 1999–2049.

Lang, N., Jetz, W., Schindler, K., & Wegner, J. D. (2023). A high-resolution canopy height model of the Earth. *Nature Ecology & Evolution*, *7*(11), 1778–1789.

Maclean, I. M. D. (2026). Microclimf: Fast modelling of microclimate across real landscapes in R. *Methods in Ecology and Evolution*, *17*(4), 1112–1123. https://doi.org/10.1111/2041-210x.70253

Raupach, M. R. (1989). A practical Lagrangian method for relating scalar concentrations to source distributions in vegetation canopies. *Quarterly Journal of the Royal Meteorological Society. Royal Meteorological Society (Great Britain)*, *115*(487), 609–632.

B - Additional figures and tables

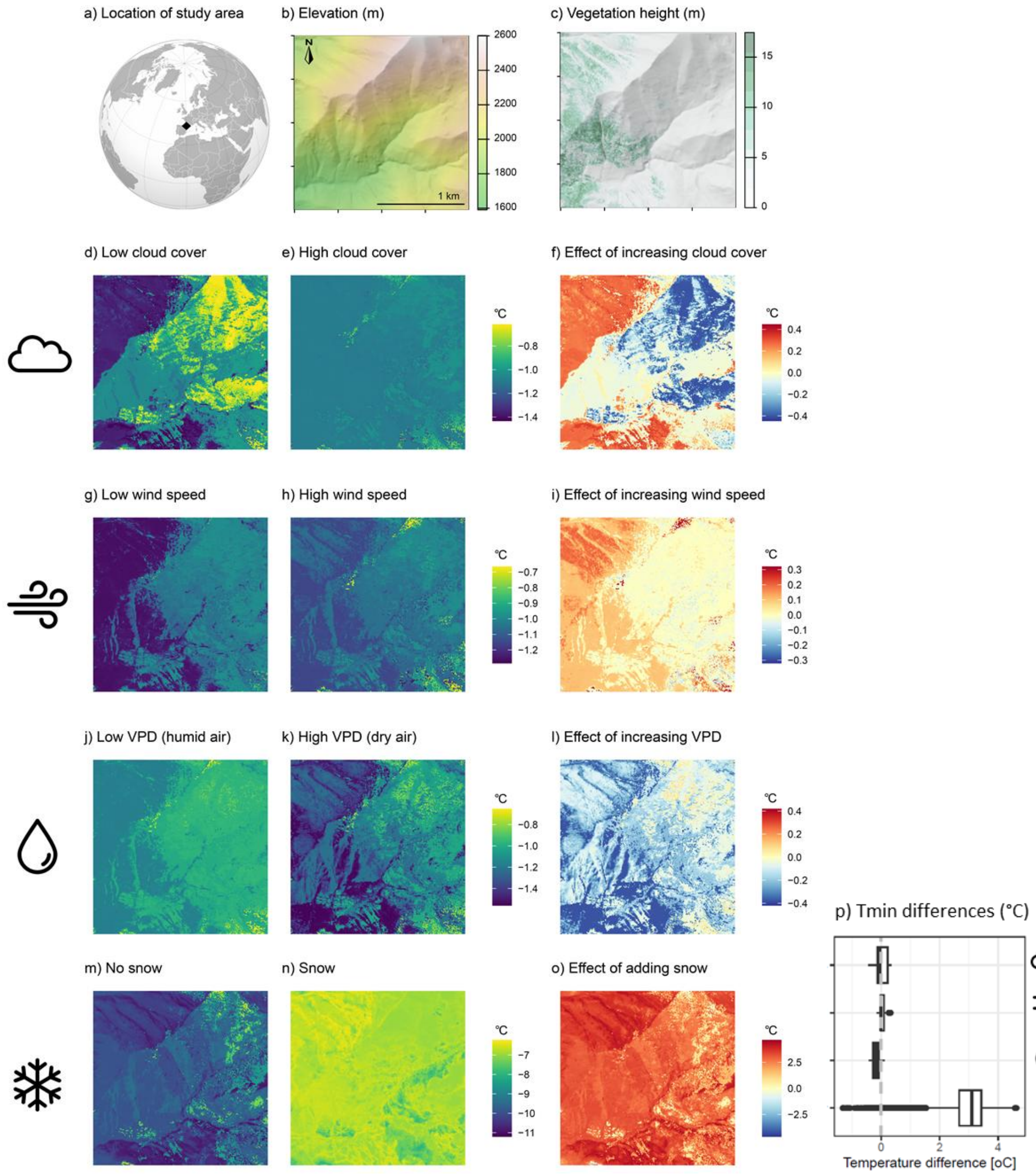

Fig. S1. Model results of case study 1 for ***minimum*** *near-surface temperature (5 cm above ground), where single meteorological variables (clouds, wind speed, air humidity, and snow cover) were modified in isolation to visualize their effect on microclimate warming and how it varies across a ca. 2 × 2 km² landscape on the border of Parque Natural Posets Maladeta in the Spanish Pyrenees (See Supporting information for more modelling details and an interpretation of observed patterns). Left and middle columns show the minimum temperature of the 10 coldest days per year when setting the respective meteorological variables at low and high values, respectively, and keeping all other variables constant. Difference maps (right column) show the differences between high and low simulated values of the meteorological variables, i.e., the effect of going from low to high values. (Icons from Flaticon.com)*

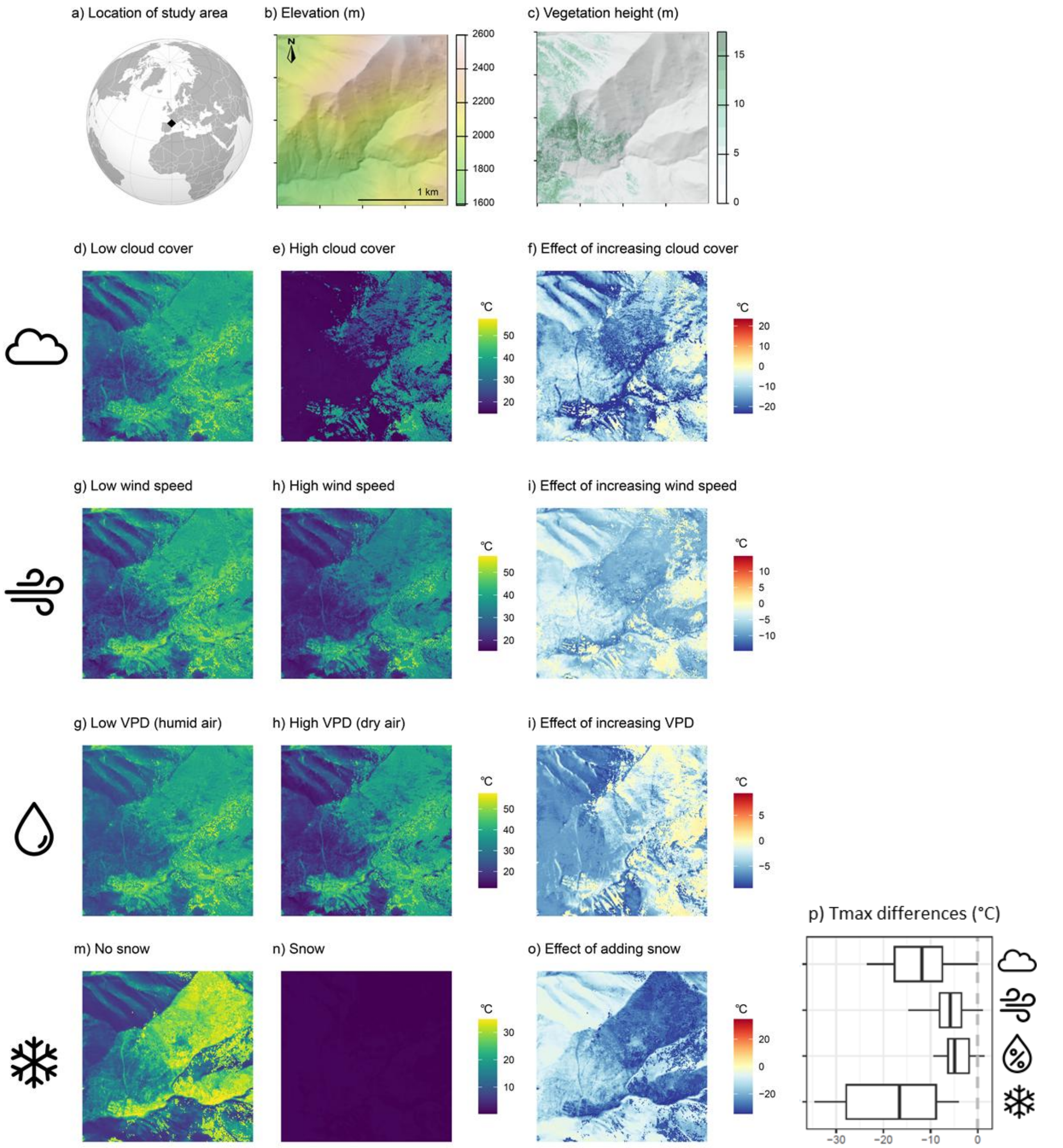

Fig. S2. Model results of case study 1 for ***maximum*** *near-surface temperature (5 cm above ground), where single meteorological variables (clouds, wind speed, air humidity, and snow cover) were modified in isolation to visualize their effect on microclimate warming and how it varies across a ca. 2 × 2 km² landscape on the border of Parque Natural Posets Maladeta in the Spanish Pyrenees (See Supporting information for more modelling details and an interpretation of observed patterns). Left and middle columns show the maximum temperature of the 10 warmest days per year when setting the respective meteorological variables at low and high values, respectively, and keeping all other variables constant. Difference maps (right column) show the differences between high and low simulated values of the meteorological variables, i.e., the effect of going from low to high values. (Icons from Flaticon.com)*

Table S1: Summary statistics of the microclimate change induced by changing atmospheric variables (cloudiness, wind speed, VPD, and snow cover). min/max = absolute minimum/maximum. q1/q3 = first/third quartile. IQR = interquartile range. SD = standard deviation.

temperature	variable	min	q1	median	mean	q3	max	IQR	SD
Tmax	clouds	-23.50	-17.64	-11.83	-12.41	-7.53	0.00	10.11	6.55
Tmax	wind	-14.68	-8.06	-5.85	-5.62	-3.45	1.01	4.61	2.98
Tmax	VPD	-9.31	-6.30	-4.86	-4.18	-1.85	1.35	4.45	2.66
Tmax	snow	-34.56	-27.84	-16.56	-18.11	-8.83	-4.08	19.01	9.69
Tmin	clouds	-0.45	-0.13	-0.04	-0.02	0.22	0.34	0.35	0.22
Tmin	wind	-0.16	-0.01	0.00	0.05	0.10	0.32	0.11	0.07
Tmin	VPD	-0.42	-0.27	-0.18	-0.19	-0.09	0.11	0.19	0.12
Tmin	snow	-1.32	2.67	3.09	2.96	3.42	4.63	0.76	0.80

Table S2: Summary statistics of the microclimate change induced by changing vegetation cover from grassland to shrub/tree dominated woodland. min/max = absolute minimum/maximum. q1/q3 = first/third quartile. IQR = interquartile range. SD = standard deviation.

variable	min	q1	median	mean	q3	max	IQR	SD
Tmax	-16.62	-13.3	-12.68	-11.93	-11.78	5.96	1.52	2.86
Tmin	-2.50	-0.5	-0.06	-0.16	0.40	1.49	0.90	0.80
